# Cross‐Sectional Comparison of Structural MRI Markers of Impairment in a Diverse Cohort of Older Adults

**DOI:** 10.1002/hbm.70133

**Published:** 2025-01-27

**Authors:** Julie K. Wisch, Kalen Petersen, Peter R. Millar, Omar Abdelmoity, Ganesh M. Babulal, Karin L. Meeker, Meredith N. Braskie, Kristine Yaffe, Arthur W. Toga, Sid O'Bryant, Beau M. Ances, the HABS‐HD Study Team

**Affiliations:** ^1^ Department of Neurology Washington University in St. Louis St. Louis Missouri USA; ^2^ Danforth Undergraduate Campus Washington University in St. Louis St. Louis Missouri USA; ^3^ Department of Family Medicine, Institute for Translational Research University of North Texas Health Science Center Fort Worth Texas USA; ^4^ Stevens Neuroimaging and Informatics Institute, Keck School of Medicine University of Southern California Los Angeles California USA; ^5^ Weill Institute for Neurosciences University of California, San Francisco San Francisco California USA

**Keywords:** Alzheimer disease, brain aging, clinical dementia rating, MRI

## Abstract

Neurodegeneration is presumed to be the pathological process measure most proximal to clinical symptom onset in Alzheimer Disease (AD). Structural MRI is routinely collected in research and clinical trial settings. Several quantitative MRI‐based measures of atrophy have been proposed, but their low correspondence with each other has been previously documented. The purpose of this study was to identify which commonly used structural MRI measure (hippocampal volume, cortical thickness in AD signature regions, or brain age gap [BAG]) had the best correspondence with the Clinical Dementia Rating (CDR) in an ethno‐racially diverse sample. 2870 individuals recruited by the Healthy and Aging Brain Study—Health Disparities completed both structural MRI and CDR evaluation. Of these, 1887 individuals were matched on ethno‐racial identity (Mexican American [MA], non‐Hispanic Black [NHB], and non‐Hispanic White [NHW]) and CDR (27% CDR > 0). We estimated brain age using two pipelines (DeepBrainNet, BrainAgeR) and then calculated BAG as the difference between the estimated brain age and chronological age. We also quantified their hippocampal volumes using HippoDeep and cortical thicknesses (both an AD‐specific signature and average whole brain) using FreeSurfer. We used ordinal regression to evaluate associations between neuroimaging measures and CDR and to test whether these associations differed between ethno‐racial groups. Higher BAG (*p*_DeepBrainNet_ *=* 0.0002; *p*_BrainAgeR_ *=* 0.00117) and lower hippocampal volume (*p =* 0.0015) and cortical thickness (*p <* 0.0001) were associated with worse clinical status (higher CDR). AD signature cortical thickness had the strongest relationship with CDR (AIC_DeepBrainNet_ = 2623, AIC_whole cortex_ = 2588, AIC_BrainAgeR_ = 2533, AIC_Hippocampus_ = 2293, AIC_Signature Cortical Thickness_ = 1903). The relationship between CDR and atrophy measures differed between ethno‐racial groups for both BAG estimates and hippocampal volume, but not for cortical thickness. We interpret the lack of an interaction between ethno‐racial identity and AD signature cortical thickness on CDR as evidence that cortical thickness effectively captures sources of disease‐related atrophy that may differ across racial and ethnic groups. Cortical thickness had the strongest association with CDR. These results suggest that cortical thickness may be a more sensitive and generalizable marker of neurodegeneration than hippocampal volume or BAG in ethno‐racially diverse cohorts.

## Graphical abstract

We compared brain age gap (BAG), hippocampal volume, and cortical thickness and their correspondence with Clinical Dementia Rating (CDR) in 629 Mexican American (MA), 629 Non‐Hispanic Black (NHB), and 629 Non‐Hispanic White (NHW) participants, finding that cortical thickness demonstrated the strongest association with dementia, and was generalizable across ethno‐racial groups.

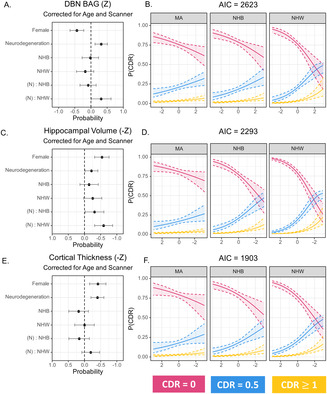

**Summary**

- Weak correlations exist between brain age gap, hippocampal volume, and cortical thickness, indicating that they likely capture complementary components of neurodegeneration.
- Non‐specific co‐pathologies (e.g., vascular brain injury) may contribute to atrophy in the cortex.
- Alzheimer Disease signature cortical thickness may be a more sensitive and generalizable marker of neurodegeneration than hippocampal volume or BAG in ethno‐racially diverse cohorts.

## 1 Introduction

Neurodegeneration is presumed to be the pathological process most proximate to clinical symptoms in Alzheimer Disease (AD) (Barkhof and Knopman 2023; Jack et al. 2024). Although not the primary intervention target in the current environment of anti‐amyloid and anti‐tau treatment trials, structural MRI is routinely collected to assess brain structure and screen for potential interventional complications (Alves, Kalinowski, and Ayton 2023). Further, the Alzheimer's Association Revised Criteria for Diagnosis and Staging of AD cites anatomic MRI as a biomarker of non‐specific neurodegeneration (Jack et al. 2024, 2018). A key goal in the field of AD research is to develop validated biomarkers that can be used to diagnose and monitor the progression of the disease. Importantly, these biomarkers must be generalized to the entire population. However, many research cohorts are primarily composed of highly educated non‐Hispanic White individuals (Weiner et al. 2023). To ensure research efforts generalize, evaluation of biomarker performance in underrepresented populations is vital.

Clinical symptoms for AD are routinely assessed via the Clinical Dementia Rating (CDR) (Morris 1997). This semi‐structured interview process involves the use of an informant to assess changes in individual behavior in six domains: Memory, orientation, judgment and problem‐solving, community affairs, home and hobbies, and personal care (Morris 1997), with an emphasis on memory. The aim of the CDR is to detect and quantify impairment that interferes with daily life in a way that is both clinically meaningful and agnostic to differences in education level, native language, or sociocultural influences (Morris 1997). Even though CDR was developed to reflect within‐individual changes and thus should not be influenced by sociocultural differences, racial disparities in CDR assessments have been documented (Kiselica et al. 2023; Steenland et al. 2010; Perales‐Puchalt et al. 2021). It has been proposed that the use of actuarial standards (i.e., relying on more information from neuropsychological testing) in assessing CDR in diverse populations may reduce disparities.

Disparities in brain structure by ethno‐racial identity have also been reported. For example, hippocampal volumes have been observed to be smaller in Black individuals than in non‐Hispanic White individuals (Dumornay et al. 2023; Zahodne et al. 2023; Hatzenbuehler et al. 2022; Meeker et al. 2021; Dickerson et al. 2009; Fotenos, Snyder, Girton, Morris, and Buckner 2005; Lockhart and DeCarli 2014). These differences were associated with structural and social determinants of health. The experience of structural stigma and racial and ethnic discrimination across major life domains, such as employment and housing, have been associated with smaller hippocampal volumes (Zahodne et al. 2023; Hatzenbuehler et al. 2022). Differences in cortical thickness between Black and White participants have also been observed. These observed racial disparities in thickness are also partially explained by measures of socioeconomic status and experiences of racism (Meeker et al. 2021; Turney et al. 2023; Hunt et al. 2021).

Although associations between race and ethnicity and health outcomes are numerous, ethno‐racial identity is a social construct that is intertwined with many factors including genetic ancestry, social determinants of health, culture, and influences of structural and institutional access or discrimination (Flanagin, Frey, and Christiansen 2021; Adkins‐Jackson et al. 2022, 2023; Babulal et al. 2019). Using ethno‐racial identity as a covariate in a model may obscure the root cause of any observed differences that researchers would be inclined to ascribe to this social construct (Adkins‐Jackson et al. 2022). Thus, we aim to identify markers of neurodegeneration that correspond with dementia symptomology in a way that is not modified by the racial identity of the study participant.

Crucially, these commonly employed biomarkers (hippocampal volume and AD signature cortical thickness) of neurodegeneration were developed to quantify brain structures in signature regions of AD‐related atrophy in predominantly non‐Hispanic White cohorts. Alternatively, it may be useful to consider MRI‐based biomarkers that more generally describe whole brain health. This approach may be particularly valuable when evaluating dementia‐related atrophy in ethno‐racially diverse cohorts, considering that there are differences in the frequency of dominant causes of dementia depending on ethno‐racial identity (Cheng et al. 2020; Bogoian and Dotson 2022; Fitten, Ortiz, and Pontón 2001; Johnson et al. 2019; Meeker et al. 2024). As such, we will evaluate both average whole cortex thickness and brain age gap (BAG) as potential whole‐brain measures of atrophy.

BAG quantifies the difference between an individual's chronological age and the model‐predicted age based on MRI features. An individual with a positive BAG is considered to have a brain appearing older than one would expect based on chronological age. This non‐specific marker of brain structure has been linked with a wide variety of clinical symptoms, including cognitive impairment (Gaser et al. 2013; Millar, Gordon, Luckett, et al. 2023; Millar, Gordon, Wisch, et al. 2023; Jirsaraie et al. 2023; Frizzell et al. 2022; Kaufmann et al. 2019). BAG has been successfully used to identify deviations from typical brain structure in neurological conditions that do not have a specific disease phenotype (Franke and Gaser 2019; Wen et al. 2024), as well as impacted by both genetic and lifestyle contributions (Wen et al. 2024; Ning et al. 2020).

As with hippocampal volume and AD signature cortical thickness, these BAG models have been developed in predominantly non‐Hispanic White training datasets (Bashyam et al. 2020; Cole and Franke 2017). However, because they are a more general measure of brain health, they may still be more sensitive than hippocampal volume or AD signature cortical thickness for identifying non‐AD pathological processes and non‐specific co‐pathologies contributing to decline in ethno‐racially diverse cohorts (Millar, Gordon, Luckett, et al. 2023; Millar, Gordon, Wisch, et al. 2023). We hypothesize that a general whole‐brain measure of brain aging may serve as a generalizable marker of structural changes associated with cognitive decline in ethno‐racially diverse cohorts. In addition to BAG, we will also evaluate the utility of whole‐brain cortical thickness (as opposed to the more targeted AD signature cortical thickness) as a whole‐brain measure of structural brain health.

In sum, it is of vital importance to the field to identify neuroimaging‐based atrophy markers that are closely tied to cognitive outcomes across diverse populations. A wide range of structural brain measures are employed in the field, without consensus on the optimal measure for neurodegeneration related to dementia. Further, both clinical status (specifically dementia status) and measures of brain health may be influenced by structural and social determinants of health. Ideally, a neurodegeneration biomarker would correspond with dementia due to AD, independent of sociocultural factors. This biomarker would be generalizable to community‐based cohorts, which are more ethno‐racially diverse and feature a greater prevalence of comorbidities. Thus, our objective was to identify the structural MRI measure that had the best correspondence dementia status in an ethno‐racially diverse sample.

## 2 Methods

This cross‐sectional study compared measures of brain health derived from structural MRI to CDR in the Health and Aging Brain Study—Health Disparities (HABS‐HD) cohort (*N* = 2870). HABS‐HD uses community‐based participatory research practice to recruit individuals who self‐report their ethno‐racial identity as Mexican American (MA), Non‐Hispanic Black (NHB), and Non‐Hispanic White (NHW) (O'Bryant et al. 2011, 2021). Enrollment criteria have been previously published (O'Bryant et al. 2021). In short, participants in HABS‐HD enroll in a 48–60 month longitudinal study that routinely conducts interviews and collects cognitive and demographic data, plasma, MRI, and PET imaging, although our current study only relies on baseline data (O'Bryant et al. 2021). Cognitive testing and interviews are conducted in either English or Spanish, at the participant's discretion. This study was approved under the North Texas Regional Institutional Review Board (#2016‐128) and the Washington University Institutional Review Board (#202311192), and all participants provided written informed consent.

### 2.1 Clinical Assessment

Clinical Dementia Rating (CDR) entails an interview with both the participant and their informant (Weiner et al. 2023; O'Bryant et al. 2011). Participants in HABS‐HD can elect to complete this clinical assessment in either English or Spanish. The CDR score summarizes the level of dementia or impairment in everyday activities. A CDR of 0 indicates no impairment. A CDR of 0.5 indicates very mild cognitive impairment (MCI). A CDR of 1 or greater indicates dementia, with increasing scores reflective of increasing severity of clinical impairment. The administrator of the CDR assessment is blind to any neuropsychological test results. Of note, participants having the most severe assessment of CDR = 3 (*N* = 8) were not included in the analysis, as it was deemed unlikely that these participants could reliably tolerate an imaging session due to increased head motion.

Because racial disparities in CDR assessments have been documented, and recommendations to include neuropsychological test results have been proposed as the remedy (Kiselica et al. 2023; Steenland et al. 2010; Perales‐Puchalt et al. 2021), we seek to validate CDR assessments across ethno‐racial groups by comparing CDR status with performance on the Preclinical Alzheimer's Cognitive Composite (PACC), an aggregation of cognitive tests designed to detect changes associated with preclinical AD (Donohue et al. 2019). If individuals, stratified by ethno‐racial identity, perform similarly on the PACC at a given CDR status, we will interpret this as a consistent application of CDR across groups. In contrast, if there are differences in PACC performance by ethno‐racial identity, we will interpret this as a biased application of CDR.

### 2.2 Neuropsychological Testing

The Preclinical Alzheimer's Cognitive Composite (PACC) was developed to detect cognitive changes associated with amyloid presence (Donohue et al. 2019; Papp et al. 2017). To calculate the PACC, we first normed scores on the following five cognitive tests: Mini‐Mental State Examination (MMSE), Animal Fluency Test, Spanish‐English Verbal Learning Test, the Digit Symbol test, and the Logical Memory II Delayed Recall test, using means and standard deviations for only cognitively normal participants. We then calculated the average of these five *Z*‐scores to determine each individual's PACC score. Participants completed all five tests.

### 2.3 MR Imaging

MRI was collected via 3T Siemens scanner (either SKYRA or VIDA) (Papp et al. 2017; O'Bryant et al. 2022). T1‐weighted magnetization‐prepared rapid gradient‐echo MR images were acquired in sagittal orientation for 176 slices. SKYRA parameters were as follows: 2300 ms repetition time, 2.93 ms echo time, 9^o^ flip angle, 1.1 × 1.1 × 1.2 mm^3^ voxel resolution. VIDA parameters were as follows: 2300 ms repetition time, 2.98 ms echo time, 9^o^ flip angle, 1.0 × 1.0 × 1.0 mm^3^ voxel resolution. FreeSurfer segmentation (Version 5.3) of the T1‐weighted whole brain volume image was performed to calculate regional cortical thickness. We calculated mean FreeSurfer‐derived AD signature cortical thickness within the Jack et al. meta‐ROI, which included bilateral entorhinal cortex, and fusiform, inferior, and middle temporal gyri (O'Bryant et al. 2021, 2022; Jack et al. 2019). We also calculated the average cortical thickness for all bilateral grey matter regions, which we will refer to as average whole cortex thickness. Whole brain cortical thickness was calculated as the average of all Z‐scored cortical thicknesses. If multiple regions were missing due to QC failure, these regions were not included in the calculation of the average. Hippocampal volume and intracranial volume were derived using HippoDeep (Thyreau et al. 2018), and hippocampal volume was corrected for intracranial volume using residuals. All segmentations were visually checked for quality using a standard protocol. All raters achieved sufficient reliability (Cohen's *κ* ≥ 0.60) to a known test set before evaluating segmentations. Hippocampal volume and AD signature cortical thickness were both corrected for scanner and then *Z*‐scored for statistical analysis. Missing values are due to QC failure; a full count of all missing values is available in Supplemental Table 2.

T1‐weighted images were also processed for estimates of brain age using two publicly available pipelines, DeepBrainNet (DBN) (https://github.com/vishnubashyam/DeepBrainNet) (Bashyam et al. 2020) and BrainAgeR (https://github.com/james‐cole/brainageR) (Cole and Franke 2017). Before the estimation of brain age with DBN, T1 images were minimally pre‐processed using ROBEX for skull stripping and linear alignment. ROBEX output was visually examined and scans failing this step were removed (*N* = 175 out of 4446 total longitudinal scans). In all cases, if the baseline scan failed visual quality control, we replaced the scan (and corresponding visit information) using a follow‐up visit. Then 80 axial slices of brain were supplied to each of the two pipelines to obtain estimates of brain age using the pre‐trained convolutional neural network.

BrainAgeR pre‐processing steps are built into the publicly available containerized solution. We supplied raw T_1_‐weighted images as inputs. The integrated pre‐processing pipeline performed tissue segmentation and spatial normalization with SPM12. Normalized images were vectorized. The grey matter, white matter, and cerebrospinal fluid were masked and combined, then supplied to the previously trained Gaussian process regression.

We then estimated BAG by subtracting the participant's age at the scan from the estimated brain age output by each of the two pipelines. We corrected both BAG estimates for age‐related bias and scanner differences before analysis, consistent with current best practice (de Lange and Cole 2020). To do this, we performed linear regression first with the BAG estimate as the dependent variable and scanner as the independent variable, then used residuals to correct BAG for scanner differences. We then performed a second linear regression with scanner‐corrected BAG estimate as the dependent variable and age as the independent variable and corrected BAG for age‐related bias using residuals.

### 2.4 Statistical Analysis

Because the purpose of this study was to examine the relationship between measures of brain structure and CDR across ethno‐racial identities, we restricted participants so there were an equal number of individuals with CDR assessments of 0, 0.5, 1, and 2 across three self‐identified groups of ethnoracial identity (MA, NHB, NHW). This was done to simplify the interpretation of the probability estimates generated by the ordinal regressions we performed. After downsampling to the matched cohort, we compared demographic characteristics across ethnoracial groups using chi‐squared tests for categorical variables (gender, education, CDR, scanner) and ANOVA for continuous variables (age).

Next, we compared the PACC score by CDR status and ethno‐racial identity. We corrected PACC for years of education based on the following categorizations: Less than high school diploma, high school diploma, some college, college degree, more than college degree. Because of concerns about differences in educational attainment as stratified by racial identity in this cohort, we completed two supplemental analyses, one using PACC without adjusting for education, and one using a two‐step correction: we first performed linear regression within the NHB cohort only (as the NHB cohort had the most uniform distribution of educational attainment of the three groups), and then we applied the estimated effect of educational attainment to adjust PACC scores for all participants. Then we performed ANOVA followed by Tukey post hoc tests with PACC score as the dependent variable and the interaction between CDR status and racial identity as the independent variables. We also used Cohen's d to estimate the effect size of ethno‐racial identity on PACC performance, stratified by CDR.

Before any analysis of brain structure (via BAG, hippocampal volume [with or without adjustment for intracranial volume]), AD signature cortical thickness, or average whole cortex thickness, we corrected the variables for both age and scanner, using residuals from linear regressions with neuroimaging measure as the dependent variable and age and scanner as independent variables. In the supplement, we also present analyses including correction for educational attainment across the entire cohort as well as correction based on the impacts of educational attainment in the NHB cohort specifically. We *Z*‐scored all neurodegeneration measures. Then we compared the correlation between each marker of neurodegeneration, stratified by ethno‐racial identity using Pearson correlations. We did this separately for cognitively normal (CDR = 0) and cognitively impaired (CDR > 0) individuals. We generated 95% confidence intervals for these correlations. We also compared estimates of BAG by the two methods, performing linear regression with BAG_BrainAgeR_ as the dependent variable and BAG_DBN_ as the independent variable. We calculated the mean average error (MAE) for each BAG in years, where a perfectly accurate model would estimate every individual's Brain Age to match his/her chronological age. Then we performed linear regressions, with each of the four neurodegeneration markers (BAG_DBN_, BAG_BrainAgeR_, hippocampal volume, and cortical thickness) as dependent variables and the interaction between race and CDR as the independent variables. We also included gender as a covariate of non‐interest. We performed post hoc Tukey tests for group differences by the same interaction between racial identity and CDR, adjusting p‐values for multiple comparisons using Bonferroni correction.

Finally, we performed ordinal regression with CDR as an ordered dependent variable (where individuals were classified as CDR = 0, 0.5, or ≥ 1) and the interaction between ethno‐racial group and neurodegenerative measure as the independent variables. We present additional analyses where the neurodegenerative measures of interest have been adjusted for educational attainment in the supplement. We also included gender as a covariate of non‐interest. To assess the goodness of fit for each marker of neurodegeneration, we compared models on the basis of the Akaike Information Criterion (AIC). We also constructed 95% confidence intervals for the probability of each CDR diagnosis by racial identity as a function of *Z*‐scored neurodegeneration marker using a 1000 iteration bootstrap method. All analyses were performed in R version 4.4.0.

## 3 Results

From the original cohort of 2870 individuals (Table S1), we identified a cohort of 1887 individuals, equally divided by both racial identity (629 MA, 629, NHB, 629 NHW) and CDR Status (72% CDR = 0, 23% CDR = 0.5, 4% CDR ≥ 1). Although now matched on CDR, these groups differed significantly on other characteristics (Table 1). The MA cohort included a greater proportion of female participants (71.7%) than the NHB cohort (66.6%), which included more female participants than the NHW cohort (60.3%). NHW participants were significantly older (mean age = 68.1) than either of the other two groups (mean age, MA = 62.7; mean age, NHB = 62.5). NHB and NHW participants had received significantly higher levels of educational attainment than the MA participants. Due to the timing of the extension of the original grant, which focused on recruitment of MA and NHW, the new grant includes more NHB (and Hispanic, non‐MA participants who were not included in this study). For the new grant, most MA (68.0%) and NHW (61.2%) participants were scanned on a Siemens SKYRA, while all NHB participants were scanned on a Siemens VIDA. The remaining MA (32.0%) and NHW (38.8%) participants were also scanned on the same Siemens VIDA.

**TABLE 1:** Participant characteristics.

	Mexican American (MA)	Non‐Hispanic Black (NHB)	Non‐Hispanic White (NHW)	*p*
*N*	629	629	629	
Age	62.72 (8.35)	62.46 (7.57)	68.14 (8.57)	< 0.001
Gender (% Female)	451 (71.7%)	419 (66.6%)	379 (60.3%)	< 0.001
Education				< 0.001
No high school	202 (32.6%)	1 (0.2%)	3 (0.5%)	
Some high school	117 (18.9%)	36 (5.7%)	14 (2.2%)	
High school graduate	110 (17.8%)	112 (17.8%)	64 (10.2%)	
Some college	95 (15.3%)	196 (31.2%)	169 (26.9%)	
College graduate	59 (9.5%)	152 (24.2%)	163 (25.9%)	
Advanced degree	36 (5.8%)	132 (21.0%)	216 (34.3%)	
Clinical dementia rating (CDR)				1.000
0	455 (72.3%)	455 (72.3%)	455 (72.3%)	
0.5	147 (23.4%)	147 (23.4%)	147 (23.4%)	
1	22 (3.5%)	22 (3.5%)	22 (3.5%)	
2	5 (0.8%)	5 (0.8%)	5 (0.8%)	
Scanner (% Vida)	201 (32.0%)	629 (100.0%)	244 (38.8%)	< 0.001

### 3.1 Relationship Between PACC and CDR

Cognitively normal MA and NHW participants had significantly higher PACC scores than NHB participants (Cohen's D = 0.258, 95%CI: [0.115, 0.402]; Cohen's D = 0.268, 95%CI: [0.110, 0.426]). There were no significant differences in performance for individuals with a clinical rating of either very mild dementia (CDR = 0.5) or mild to moderate dementia (CDR ≥ 1) (Figure S1A). These patterns were similar, regardless of the method employed for handling differences in cohort education level (Figure S1B,C).

### 3.2 Relationship Between Markers of Neurodegeneration

**FIGURE 1:**
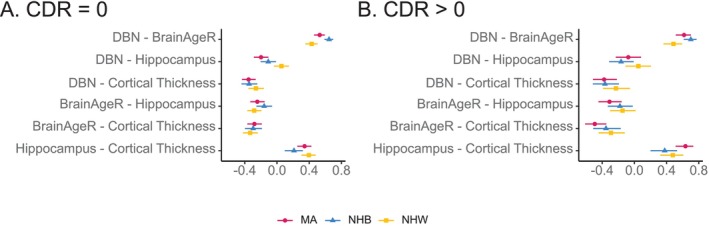
Correlations between markers of neurodegeneration. (A) Pearson correlations for comparisons between various markers of neurodegeneration are presented (Brain age gap calculated via Deep Brain Net (DBN), brain age gap calculated via BrainAgeR, hippocampal volume, and cortical thickness), stratified by racial identity, for both unimpaired (CDR 0) (A) and impaired (CDR > 0) (B) participants. The strongest correlation for unimpaired and impaired was between markers of Brain Age Gap. Relationships were generally consistent between the unimpaired and impaired for the various markers of neurodegeneration.

The strongest correlation between MRI biomarkers was between hippocampal volume, whether or not it was corrected with intracranial volume(*r*_CDR=0_ = 0.917, 95%CI: [0.907, 0.925]; *r*_CDR > 0_ = 0.885, 95%CI: [0.863, 0.903]) and AD signature cortical thickness with average whole cortex thickness (*r*_CDR=0_ = 0.807, 95%CI: [0.786, 0.827]; *r*_CDR > 0_ = 0.862, 95%CI: [0.833, 0.886]). The two BAG calculation methods were also relatively highly correlated (*r*_CDR=0_ = 0.521, 95%CI: [0.481, 0.559]; *r*_CDR > 0_ = 0.601, 95%CI: [0.542, 0.653]). All other correlations were between 0.4 and −0.4 (Figure 1). When stratified by ethnoracial identity, there were relatively few differences, although, in the cognitive normal, there was a stronger correlation between DBN and BrainAgeR in NHB (*r*_CDR=0_ = 0.647, 95%CI: [0.589, 0.699]) than NHW (*r*_CDR=0_ = 0.431, 95%CI: [0.353, 0.504]) and a stronger correlation between DBN and hippocampal volume in MA (*r*_CDR=0_ = −0.198, 95%CI: [−0.287, −0.106]) than NHW (*r*_CDR=0_ = 0.0554, 95%CI: [−0.0390, 0.149]). The relative strength of correlation between DBN and BrainAgeR persisted in the cognitively impaired participants, where NHB had a stronger correlation (*r*_CDR > 0_ = 0.701, 95%CI: [0.613, 0.771]) than NHW (*r*_CDR > 0_ = 0.485, 95%CI: [0.361, 0.593]). Detailed scatterplots of all measures, stratified by ethno‐racial identity, are available in the Supplement (Figures S2–S4). The different estimates of BAG were similar (Figures S5A and S6A), although the overall accuracy of DBN was greater than BrainAgeR, regardless of whether we evaluated the model in all participants (MAE_DBN_ = 5.10 years, MAE_BrainAgeR_ = 8.18 years; Figures S5B and S6B) or only in cognitively normal participants (MAE_DBN_ = 4.96 years, MAE_BrainAgeR_ = 7.80 years; Figures S5C and S6C).

### 3.3 Neurodegeneration Markers by Racial Identity and CDR

We compared neurodegeneration markers across both ethno‐racial identity and CDR to look for systematic differences in sensitivity to dementia by ethno‐racial identity. If systematic differences by racial identity exist, we would interpret that as the marker of neurodegeneration encompassing external factors (e.g., social determinants of health) that would otherwise be attributed to racial identity.

**FIGURE 2:**
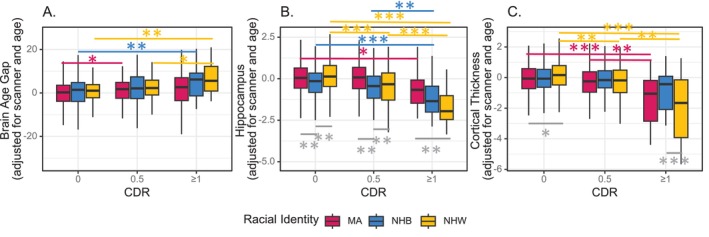
Neurodegenerative markers stratified by racial identity and Clinical Dementia Rating (CDR). *indicates *p*_adj_ < 0.05, **indicates *p*_adj_ < 0.001, ***indicates *p*_adj_ < 0.0001. Within CDR differences are indicated in grey. Within the ethno‐racial group differences are shown in their affiliated color (MA in pink, NHB in blue, NHW in yellow). For readability, differences spanning CDR and ethnoracial groups (e.g., CDR = 0 MA vs. CDR = 0.5 NHW) are omitted. (A) Brain Age Gap as calculated by Deep Brain Net increases with increasing CDR, indicating that brains appear older than chronological age in individuals with dementia. (B) Hippocampal volume declines with increasing CDR. Note that for MA, hippocampal volume does not decline until CDR ≥ 1. (C) Cortical thickness declines at the group level for MA and NHW, but not NHB.

We observed a positive relationship between BAG_DBN_ and CDR (*β*_normalized,CDR = 0.5_ = 0.200, *p* = 0.0002; *β*_normalized,CDR ≥ 1_ = 0.682, *p* < 0.0001), indicating greater cognitive impairment was associated with elevated apparent brain age. Results are highly similar for BAG_BrainAgeR_, so we report those results in supplemental materials (Figure S3). There is also a significant main effect of race for BAG_DBN_, such that BAG estimates appear almost 1 year older in NHW as compared with MA participants (*β*_normalized_ = 0.117, Figure 7A). The results of the Tukey post hoc test showed that in MA participants, BAG_DBN_ is significantly elevated in those with very mild dementia (CDR = 0.5) relative to cognitively normal (CDR = 0) participants (Difference = 2.03 years, *p*_adjusted_ = 0.027). In NHB participants, BAG_DBN_ is not elevated in those with very mild dementia (CDR 0.5, Difference = 1.67 years, *p* = 0.144), but is elevated in mild to moderate dementia (CDR ≥ 1, Difference = 5.96 years, *p*_adjusted_ = 0.0001). In NHW participants, BAG_DBN_ is not elevated in those with very mild dementia (CDR 0.5, Difference = 1.52 years, *p* = 0.248), but is elevated in mild to moderate dementia (CDR ≥ 1, Difference = 4.91 years, *p*_adjusted_ = 0.005). Overall, we observe no differences by racial identity, but also relatively few differences by CDR (Figure 2A). These results were very similar when we controlled for educational attainment (Figure S9).

Worse clinical status was associated with lower hippocampal volume (*p* = 0.002). Hippocampal volume estimates were significantly lower in NHB participants compared with MA and NHW participants (*p* < 0.0001), and there was also a significant interaction between CDR and NHW (*p* < 0.0001) such that dementia‐related differences in hippocampal volume were greater in NHW than MA participants (Figure S7B). In MA participants hippocampal volume was lower in mild to moderate dementia (CDR > =1) compared with cognitively normal participants (Difference = −0.6999σ, *p* = 0.0138), but did not differ in very mild dementia (Difference = −0.062σ, *p* = 0.999). In NHB participants, hippocampal volume was lower in mild to moderate dementia (CDR ≥ 1) compared with cognitively normal participants (CDR 0, Difference = −1.050σ, *p* > 0.0001) and participants with very mild dementia (CDR 0.5, Difference = −0.808σ, *p* = 0.002). In NHW participants, hippocampal volume was lower in very mild dementia (CDR 0.5, Difference = −0.614σ, *p* < 0.0001) and mild to moderate dementia (CDR ≥ 1, Difference = −1.989σ, *p* < 0.0001) compared with cognitively normal participants. Overall, we observe a relatively large amount of variance in hippocampal volume by both CDR status and racial identity (Figure 2B). These results were highly similar when we controlled for educational attainment, as well as when we did not control for intracranial volume (Figure S10).

Worse clinical status was also associated with lower AD signature cortical thickness (*p* < 0.0001). There was also a significant main effect of race, such that AD signature cortical thickness estimates were greater in NHW compared with MA participants (*p* = 0.002 (Figure S7C)). In MA participants AD signature cortical thickness was not lower in very mild dementia (CDR 0.5) compared with cognitively normal participants (Difference = −0.190σ, *p* = 0.694), but was lower in mild to moderate dementia (CDR ≥ 1) compared with cognitively normal participants and very mild dementia (Difference = −1.2251σ, −1.035σ, *p* < 0.0001, = 0.0003 respectively). In NHB participants, AD signature cortical thickness did not significantly differ as a function of CDR status. In NHW participants, AD signature cortical thickness was lower in both mild to moderate (CDR ≥ 1) and very mild dementia (CDR 0.5) compared with cognitively normal participants (Difference = −2.023ơ, −1.601, *p* < 0.0001, *p* < 0.0001). Overall, we observed a clear stepwise relationship between AD signature cortical thickness and CDR for both MA and NHW, but not NHB. There was also variance in cortical thickness as a function of ethno‐racial identity (Figure 2C). These results were highly similar when we controlled for educational attainment (Figure S11).

### 3.4 Probability of CDR Diagnosis

**FIGURE 3:**
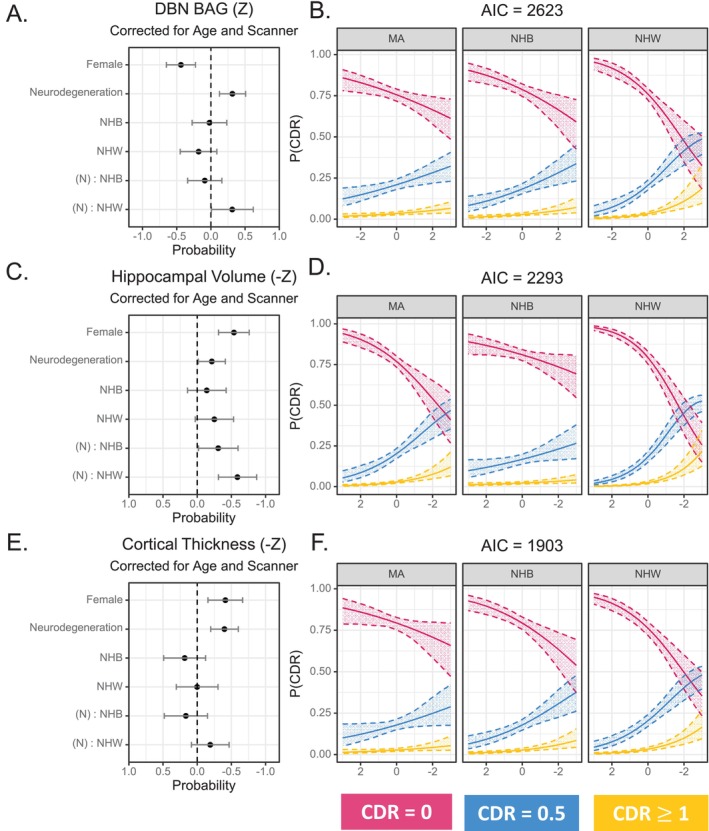
Probability of CDR as a function of neurodegenerative marker, racial identity, and gender. Mexican American (MA) participants served as the reference cohort in these analyses and, as such, are not depicted in the forest plots. (A) There are significant effects of gender, brain age gap (BAG) as calculated by Deep Brain Net (DBN), and a significant interaction between BAG and racial identity for non‐Hispanic Whites (NHW) indicating that (B) the probability of being cognitively normal (CDR = 0) declines at a greater rate with increasing BAG for NHW than either Mexican American (MA) or non‐Hispanic Black (NHB) participants. (C) There are significant effects of gender, hippocampal volume, and racial identity for NHW and significant interactions between hippocampal volume and racial identity for both NHB and NHW, indicating that (D) the probability of different CDR diagnoses is least associated with hippocampal volume for MA. The probability of being CDR > 0 increases more rapidly for NHB than MA with declining hippocampal volume. This probability increases even more rapidly for NHW relative to MA. (E) There are significant effects of gender and cortical thickness, but not racial identity, on the probability of being cognitively normal or impaired. (F) This means that although the probability of CDR diagnosis changes with decreasing cortical thickness, there are no differences by ethnoracial identity.

We applied ordinal regression to assess the utility of each neurodegeneration measure for classifying CDR status. We observed a statistically significant main effect of BAG_DBN_ (*β* = 0.314, 95% CI: [0.123, 0.506], *p* = 0.001). There was also a statistically significant interaction between BAG_DBN_ and NHW identity (*β* = 0.312, 95% CI: [0.006, 0.618], *p* = 0.0499) (Figure 3A), indicating that the association between BAG_DBN_ and CDR was greater in NHW than in either MA or NHB participants (Figure 3B). This result was not altered by controlling for education (Figure S12). Again, we observed similar results with BAG_BrainAgeR_ in (Figures S7B, S8 and S13).

We also observed a significant main effect of hippocampal volume (*β* = −0.213, 95% CI: [−0.410, −0.017], *p* = 0.0328) in relation to CDR status. There were also significant interactions between hippocampal volume and both NHB (*β* = −0.307, 95% CI: [−0.596, −0.018], *p* < 0.001) and NHW identity (*β* = −0.588, 95% CI: [−0.867, −0.309], *p* = 0.0387), indicating that the relationship between hippocampal volume and CDR was greater in NHW than in either MA or NHB participants (Figure 3C,D). There was minimal impact on the results when we controlled for education (Figure S14).

Finally, there was also a significant main effect of AD signature cortical thickness in relation to CDR status (*β* = −0.398, 95% CI: [−0.599, −0.197], *p* = 0.0001). There results were not substantially affected, regardless of how educational attainment was handled (Figure S15). This effect was larger than that observed for BAG_DBN_ and hippocampal volume, although not significantly so. There were no significant interactions between AD signature cortical thickness and racial identity (*β*_NHB_ = −0.184, 95% CI: [−0.122, 0.489], *p*_NHB_ = 0.3214; *β*_NHW_ = −0.191, 95% CI: [−0.463, 0.082], *p*_NHW_ = 0.1719) (Figure 3E,F), indicating that the relationship between AD signature cortical thickness and CDR did not differ between MA, NHB, and NHW participants.

This result was somewhat different when compared with average whole cortex thickness. The average whole cortex was significantly associated with CDR status (*β* = −0.259, 95% CI: [−0.442, −0.077], *p* = 0.0053) (Figure S11). There was also a significant interaction between average whole cortex thickness and NHW identity (*β* = −0.288, 95% CI: [−0.540, −0.036], *p* = 0.0248), indicating that the relationship between average whole cortex thickness and CDR was stronger in NHW compared with either MA or NHB participants (Figure S16). Overall, the model relying on AD signature cortical thickness to predict CDR had the best model fit based on AIC (AIC_Cortical Thickness_ = 1903; AIC_Hippocampus_ = 2293; AIC_BrainAgeR_ = 2533; AIC_whole cortex_ = 2597; AIC_DBN_ = 2623).

## 4 Discussion

We compared the concordance between measures of atrophy obtained via MRI (BAG calculated via DeepBrainNet, BAG calculated via BrainAgeR, hippocampal volume, AD signature cortical thickness, and average whole brain cortex thickness) and CDR in an ethno‐racially diverse sample matched on CDR. Generally, we observed expected trends of either greater BAG or lower hippocampal volume and cortical thickness (either AD signature‐specific or average whole brain) with increasing dementia severity. However, these relationships varied by ethno‐racial identity. All measures of brain structure were significantly associated with CDR, although cortical thickness had the strongest relationship with CDR. Hippocampal volume had the next strongest association, followed by the two measures of BAG. Our findings highlight that there are relatively few differences across ethno‐racial identity in these measures of brain structure in their relationship to CDR clinical status, although cortical thickness was slightly more generalizable than the other evaluated measures.

Given documented differences in CDR scoring by racial identity (Kiselica et al. 2023; Steenland et al. 2010; Perales‐Puchalt et al. 2021), we first compared CDR by PACC neuropsychological test performance, as stratified by racial identity. There were significant but small (by Cohen's D) differences in PACC performance by CDR stratified by ethno‐racial identity for cognitively normal individuals. In non‐impaired individuals, we did observe slightly better performance by MA and NHW individuals even after correcting for educational attainment (Picarra and Glocker 2023). Although statistically significant, this observed difference is small and these differences did not persist in individuals that were impaired. Thus, we conclude that overall, CDR has been applied in a consistent manner by ethno‐racial identity in this cohort, assuming that PACC is representative of cognitive abilities that would correspond to dementia severity. We note that there are limitations to this assumption: the PACC is designed to be sensitive to changes in performance on cognitive tests relative to group‐level norms; CDR is designed to detect within‐individual change in clinical status. However, recent work has encouraged the use of actuarial approaches (i.e., relying more heavily on neuropsychological performances) to reduce disparities in CDR assessments (Kiselica et al. 2023). Interpretation of this result should hold both things in mind.

Next, we investigated the relationship between the various structural measures. We observed relatively low correlations between various MRI markers, indicating that although these measures may all describe neurodegeneration, they likely capture complementary components of neurodegeneration. Exceptions to this were hippocampal volume with and without correction for intracranial volume and AD signature cortical thickness with average whole cortex thickness, which is why we only present one measure of hippocampal volume and one measure of cortical thickness in the primary text. Prior work has reported a relatively high (*R* = 0.64) correlation between BrainAgeR and DeepBrainNet (Bacas et al. 2023). We observe a similar correlation (*R* = 0.57 in all individuals, both cognitively impaired and unimpaired). We also observe Spearman correlations between hippocampal volume and cortical thickness on the order of 0.3–0.4, similar to previous work (Jack et al. 2014; Leuzy et al. 2019; Bucci, Chiotis, and Nordberg 2021; Mattsson‐Carlgren et al. 2020). Of note, though, is the relatively low correlation between hippocampal volume and AD signature cortical thickness specifically in the NHB participants (*R* = 0.141, Figure S3). We speculate that this reduced relationship between AD signature cortical thickness and hippocampal volume may be attributed to baseline differences in hippocampal volume that we observe here and in prior studies (Dumornay et al. 2023; Zahodne et al. 2023; Hatzenbuehler et al. 2022; Meeker et al. 2021). The functional implications of this decoupling between cortical thickness and hippocampal volume warrant further study. Overall, we conclude that these imaging markers generally relate to one another in similar ways as compared with previously published research cohorts. Each contains distinct information and it seems probable that one MRI measure would have better correspondence with CDR than others.

Thus, we compared these structural MRI measures with CDR, stratified by ethno‐racial identity. All four measures differed in the anticipated directions as a function of CDR, although differences were slightly more clear and systematic in NHW than either MA or NHB participants, as evidenced by the relatively higher frequency of significant differences between CDR levels shown in Figure 2. Importantly, we establish that in cognitively normal individuals, there are no differences in BAG by ethno‐racial identity (via either DeepBrainNet or BrainAgeR). This finding contrasts with differences that have been observed in hippocampal volume and cortical thickness between ethno‐racial groups from previous studies (Meeker et al. 2021; Turney et al. 2023). Differences in hippocampal volume and AD signature cortical thickness were replicated in the current study (Figure 2B,C). Hence, BAG might offer utility as a structural MRI biomarker in cognitively normal cohorts that are ethno‐racially diverse.

As the ultimate aim was to identify which marker of neurodegeneration had the most generalizable relationship with CDR across ethno‐racial groups, we performed ordinal regression with CDR as the dependent variable and the interaction between ethno‐racial identity and neurodegeneration biomarker as the independent variables of interest (with gender as a covariate). From this analysis, we conclude that AD signature cortical thickness has the strongest correspondence with CDR. The model using AD signature cortical thickness had the lowest AIC, indicating the best model fit. Further, there were no significant terms between cortical thickness and ethno‐racial identity. From this, we infer that the AD signature cortical signature relates to dementia status in a manner that is generalizable across ethno‐racial groups. It is notable that of all the structural MRI measures commonly reported in clinical trial outcomes, AD signature cortical thickness has not been generally utilized (although Donanemab did report bilateral cortical volume) (Alves, Kalinowski, and Ayton 2023). Given this strong association with CDR and apparent generalizability to a community‐based cohort, AD signature cortical thickness could be a valuable measure to report in future clinical trials, as we seek to understand the impact of changes in brain structure in anti‐amyloid and anti‐tau therapies.

Of note, when we compare the models derived from hippocampal volume, we observe a profoundly weaker relationship between hippocampal volume and CDR in the MA cohort (Figure 3D). There is relatively little relationship between declining hippocampal volume and increasing/decreasing probability of a specific CDR diagnosis. This suggests that the instigators of dementia in MA are not captured by hippocampal volume (Jack et al. 2018; Housini et al. 2023) Given the relatively low prevalence of both APOEε4 carriership (Housini et al. 2023) and amyloid positivity (Meeker et al. 2024) in MA participants in this cohort, MA individuals may be more likely to suffer from non‐AD dementia or mixed pathology dementia, both of which may have less impact on the hippocampus. We did consider that the relatively lower educational attainment of the MA participants in this study may be influencing the relationship between structural MRI measures and CDR; however, our supplemental analyses indicate that the observed relationships were robust, regardless of whether or not we controlled for education.

In contrast, we observed consistently good correspondence between AD signature cortical thickness and CDR, regardless of ethnoracial identity. While we cannot definitively determine the reason why AD signature cortical thickness is the best imaging proxy for CDR in this diverse cohort, we note that a recent study observed a relationship between cortical thinning and the presence of vascular disease, independent of amyloid (Keuss et al. 2024). If vascular disease is one of the key drivers of dementia in MA and NHB individuals (Cheng et al. 2020; Bogoian and Dotson 2022; Fitten, Ortiz, and Pontón 2001; Johnson et al. 2019), it is possible that AD signature cortical thickness may capture these changes. It also could be that AD signature cortical thickness regions cover a greater proportion of the brain than hippocampal volume, and thus may have a greater signal‐to‐noise ratio and/or greater sensitivity to detect non‐AD associated atrophy. Although the greater area covered by the AD signature cortical thickness may lead to its effectiveness, it did outperform multiple whole‐brain measures, suggesting that some level of specificity is important. The AD signature cortical thickness outperformed the average whole cortex as well as BAG. Although BAG is informed by whole‐brain imaging data, AD signature cortical thickness was developed specifically to track changes associated with AD, while BAG tracks normative aging. The AD signature cortical thickness may represent a compromise in detecting dementia‐relevant atrophy by including a sufficient proportion of the brain to be informative without capturing nonspecific brain changes. Alternatively, it may also be possible that non‐specific co‐pathologies (e.g., vascular brain injury) may have contributed to atrophy in the original selection of the AD signature regions, and thus, these regions may not reflect a “pure” AD signature.

Overall, AD signature cortical thickness has the strongest model fit with CDR status and has no significant interaction terms with ethno‐racial identity. Based on this observation, we recommend employing AD signature cortical thickness rather than hippocampal volume as a structural correlate of dementia, particularly in studies involving ethno‐racially diverse cohorts. Our work suggests that AD signature cortical thickness has stronger correspondence with clinical status, CDR, even in entirely non‐Hispanic White cohorts, but also that it is more generalizable across ethno‐racial groups. Further, although we considered general measures of brain health like whole cortex thickness and BAG as a potential remedy for sensitivity to non‐AD contributions to dementia, we find that in this cohort, both these measures are less sensitive to dementia status than either hippocampal volume or cortical thickness.

This study was limited in that it is cross‐sectional in nature and included relatively few individuals with severe dementia. Future work as longitudinal data becomes available in the HABS‐HD cohort could determine if the rate of decline in different regions is more or less predictive of changes in CDR status. Further, additional investigation of markers that describe other causes of degeneration like vascular dementia (e.g., white matter hyperintensities, Framingham risk score) could be of great value in this community‐based sample. Future analyses once APOEε4 carriership, a known genetic risk factor for AD, is available for all participants would also be informative.

## 5 Conclusion

Our objective was to identify the structural MRI measure that had the best correspondence with CDR in an ethno‐racially diverse sample. We observed relatively low correlations between BAG, hippocampal volume, and cortical thickness, suggesting that each neuroimaging measure captures distinct aspects of brain structure and neurological health. We observed that the AD signature cortical thickness signature demonstrated the strongest association with dementia status, and was relatively generalizable across ethno‐racial groups. In conclusion, out of the measures evaluated here, we recommend using AD signature cortical thickness as a biomarker of dementia‐related atrophy when evaluating ethno‐racially diverse cohorts.

## Conflicts of Interest

B.M.A receives research funding from the National Institutes of Health and has a patent (“Markers of Neurotoxicity in CAR T patients”). All other authors declare no competing interests.

## Supporting information


Data S1.

## Data Availability

The data used in this analysis are available on request at https://apps.unthsc.edu/itr/researchers.
