# Supplementary material for: Cross‐Sectional Comparison of Structural MRI Markers of Impairment in a Diverse Cohort of Older Adults
Source: Hum Brain Mapp. 2025 Jan 27;46(2):e70133. doi: 10.1002/hbm.70133 (PMC11770891; doi:10.1002/hbm.70133)
Supplement: Supplementary file 1 — Data S1. [file HBM-46-e70133-s001.docx]

**SUPPLEMENTAL MATERIALS**

**Supplemental Table 1. Original cross-sectional cohort.** We downsampled from this cohort by matching on CDR and removing all individuals of CDR = 3 to generate our cohort for analysis.

|  | **Mexican American (MA)** | **Non-Hispanic Black (NHB)** | **Non-Hispanic White (NHW)** | **p** |
| --- | --- | --- | --- | --- |
| N | 1028 | 667 | 1043 |  |
| Age | 62.95 (8.02) | 62.77 (7.74) | 68.40 (8.67) | <0.001 |
| Gender (% Female) | 717 (69.7%) | 440 (66.1%) | 629 (60.3%) | <0.001 |
| Education |  |  |  | <0.001 |
| No High School | 358 (35.4%) | 1 (0.1%) | 4 (0.4%) |  |
| Some High School | 198 (19.6%) | 36 (5.4%) | 26 (2.5%) |  |
| High School Graduate | 168 (16.6%) | 123 (18.4%) | 114 (10.9%) |  |
| Some College | 143 (13.1%) | 209 (31.3%) | 276 (26.5%) |  |
| College Graduate | 94 (9.3%) | 138 (20.7% | 229 (32.5%) |  |
| Advanced Degree | 50 (4.9%) | 138 (20.7%) | 339 (32.5%) |  |
| Clinical dementia rating (CDR) |  |  |  | <0.001 |
| 0 | 762 (74.1%) | 455 (68.2%) | 863 (82.7%) |  |
| 0.5 | 224 (21.8%) | 177 (26.5%) | 147 (14.1%) |  |
| 1 | 33 (3.2%) | 22 (3.3%) | 25 (2.4%) |  |
| 2 | 8 (0.8%) | 9 (1.3%) | 5 (0.5%) |  |
| 3 | 1 (0.1%) | 4 (0.6%) | 3 (0.3%) |  |
| Scanner (% Vida) | 218 (21.2%) | 667 (100%) | 295 (28.3%) | <0.001 |

**Supplemental Table 2. Summary of Missing Data.** Because of quality control failures, not all values were available for all participants.

|  | **Mexican American (MA)** | **Non-Hispanic Black (NHB)** | **Non-Hispanic White (NHW)** | **p** |
| --- | --- | --- | --- | --- |
| Original N | 629 | 629 | 629 | 1.000 |
| Missing BAG - DeepBrainNet | 0 | 0 | 0 | 1.000 |
| Missing BAG - BrainAgeR | 14 (2.2%) | 32 (5.1%) | 15 (2.4%) | 0.006 |
| Missing AD Signature Cortical Thickness | 91 (14.5%) | 206 (32.8%) | 137 (21.8%) | < 0.001 |
| Missing Average Whole Cortex Thickness | 3 (0.5%) | 3 (0.5%) | 3 (0.5%) | 1.000 |
| Missing Hippocampal Volume | 43 (6.8%) | 60 (9.5%) | 43 (6.8%) | 0.117 |


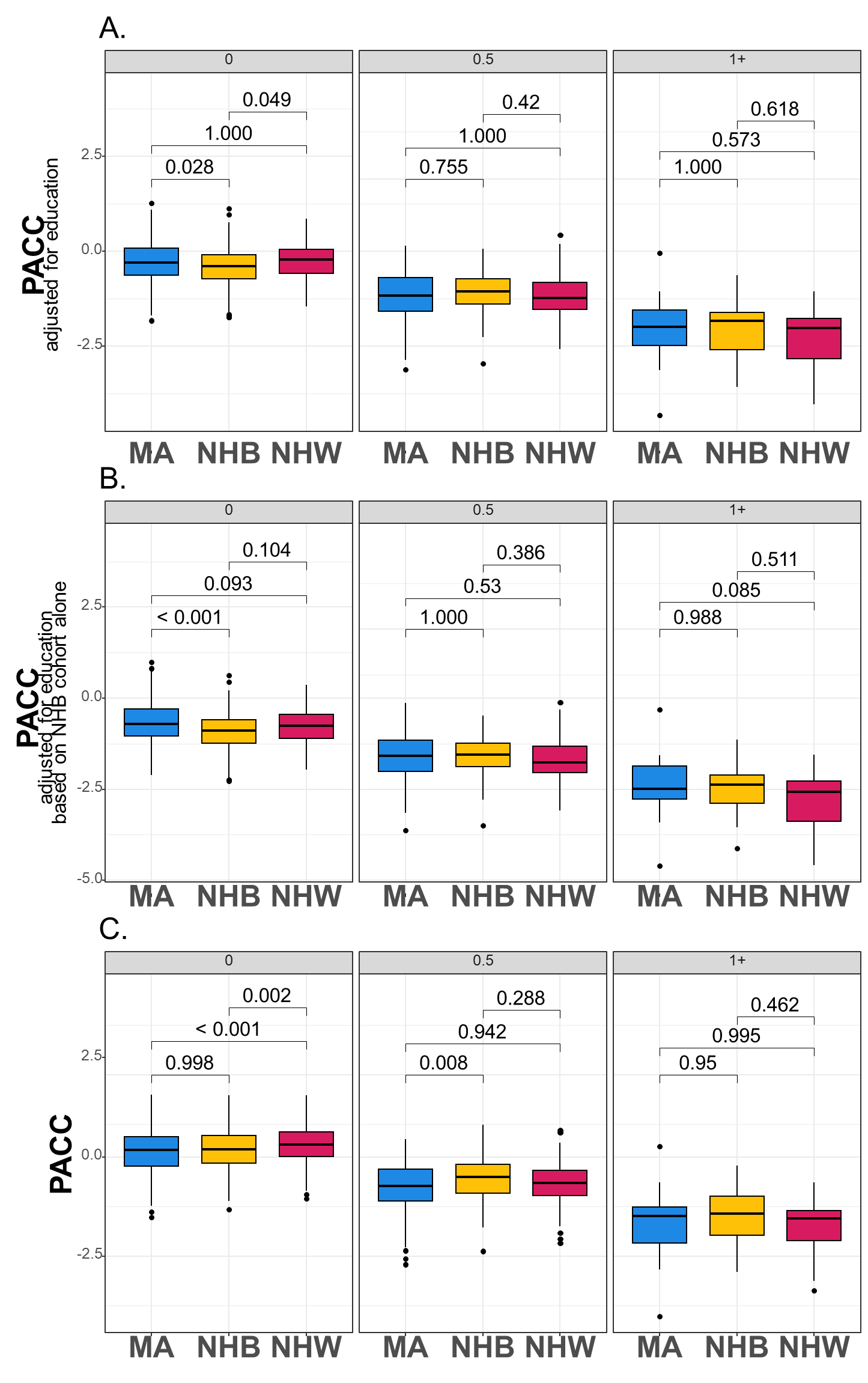


**Supplemental Figure 1. Comparison of Preclinical Alzheimer Cognitive Composite (PACC) performance as stratified by Clinical Dementia Rating (CDR) and Racial Identity (MA: Mexican American, NHB: Non-Hispanic Black, NHW: Non-Hispanic White).** We considered potential methods of adjusted the PACC, adjusting for educational attainment (1A), adjusting for educational attainment based on the differential impact of educational attainment in the NHB cohort (1B), not adjusting for educational attainment (1C). In all cases, significant differences were observed across ethno-racial group for CDR = 0 individuals, but there were no differences by ethnoracial group in individuals with either very mild impairment (CDR = 0.5) or impairment (CDR ≥ 0).


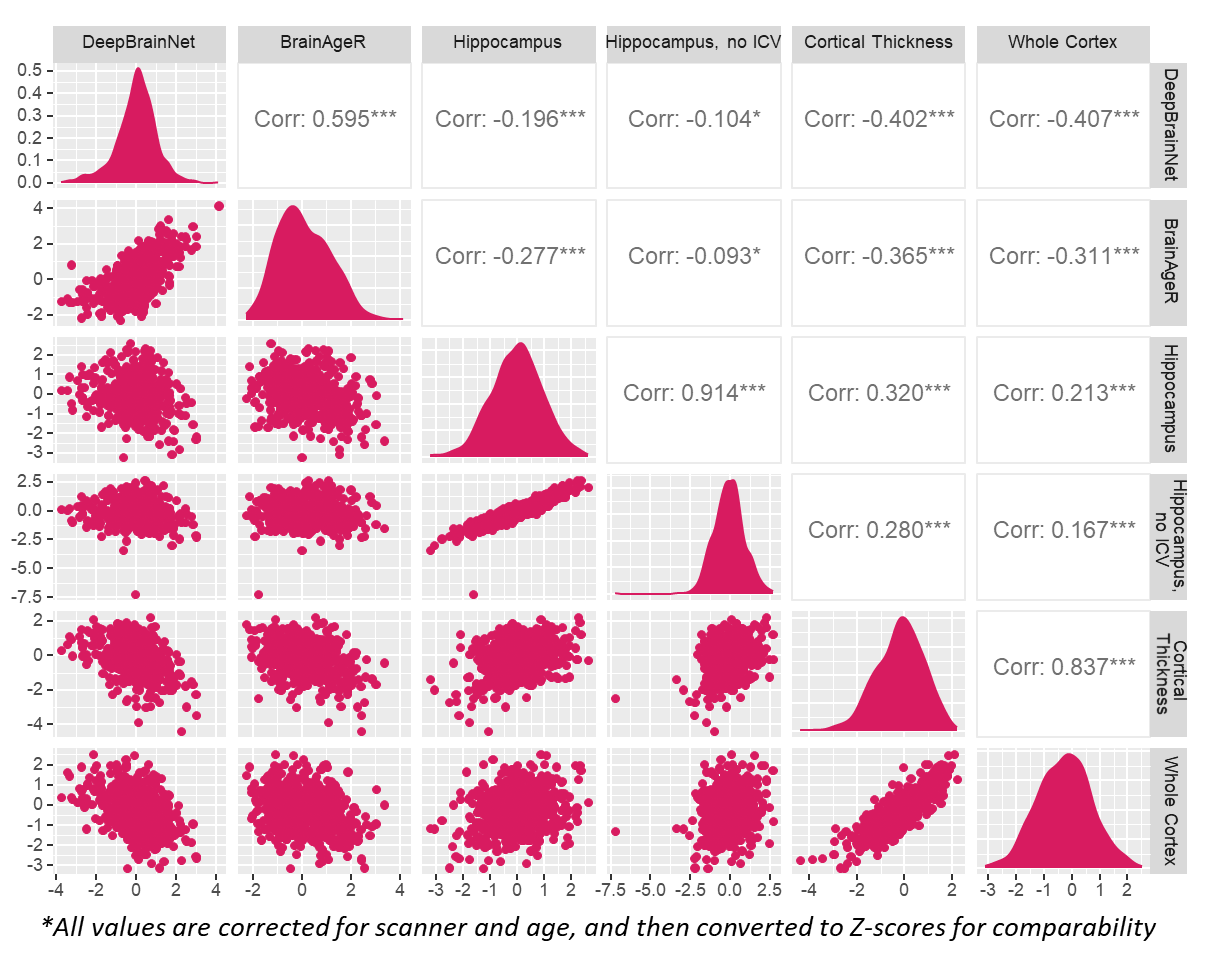


**Supplemental Figure 2**. Comparison of markers of neurodegeneration in Mexican American participants. Hippocampal volume with and without intracranial volume (ICV) correction have the strongest correlation, followed by AD signature cortical thickness and average whole cortex thickness. The third-strongest correlation is between methods of Brain Age Gap (BAG) calculation, followed by Cortical Thickness and BAG_Deep Brain Net_.


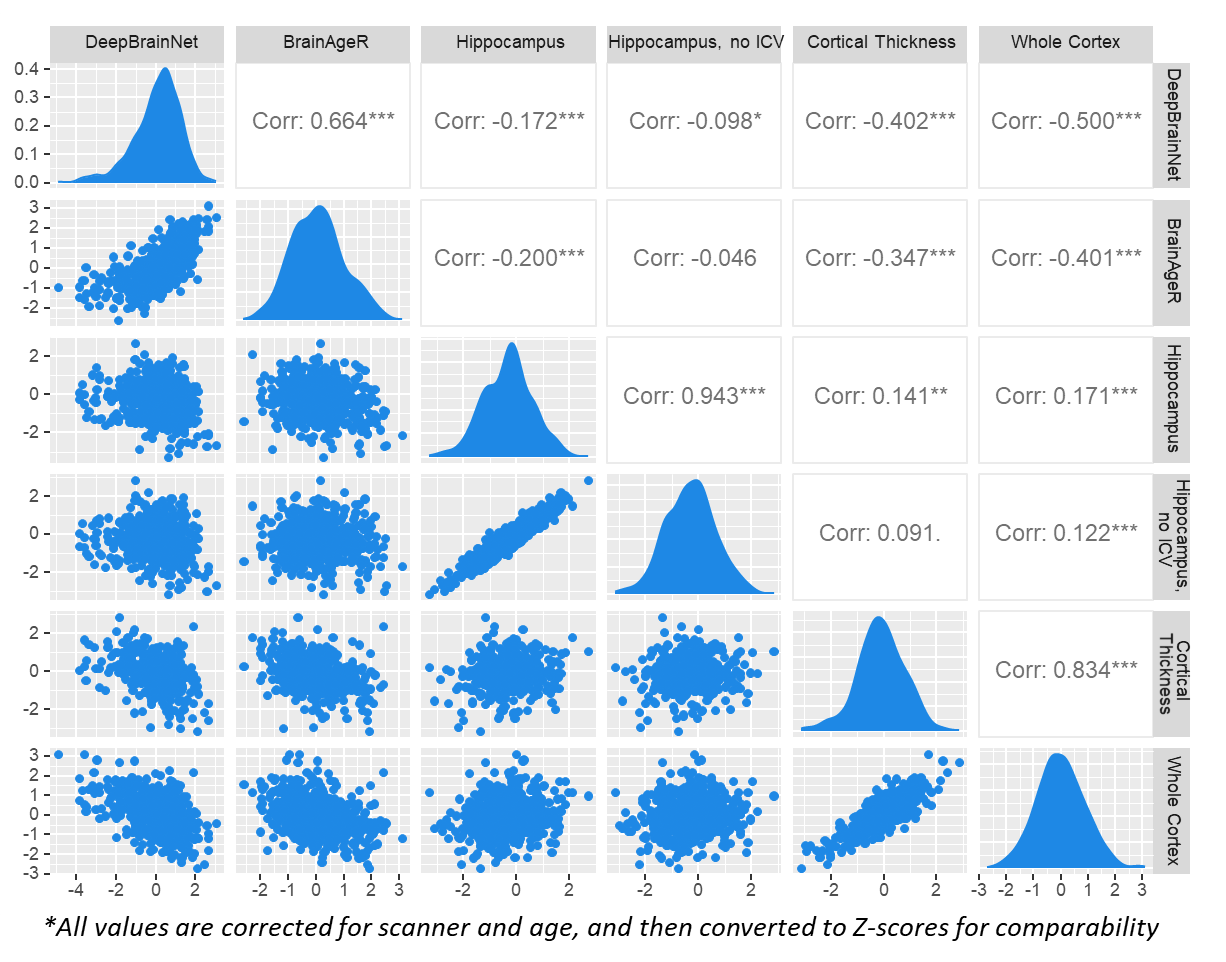


**Supplemental Figure 3**. Comparison of markers of neurodegeneration in Non-Hispanic Black participants. Hippocampal volume with and without intracranial volume (ICV) correction have the strongest correlation, followed by AD signature cortical thickness and average whole cortex thickness. The third strongest correlation is between methods of Brain Age Gap (BAG) calculation, followed by Cortical Thickness and BAG_Deep Brain Net_. The correlation between cortical thickness and hippocampal volume is much lower than is typically reported in the literature.


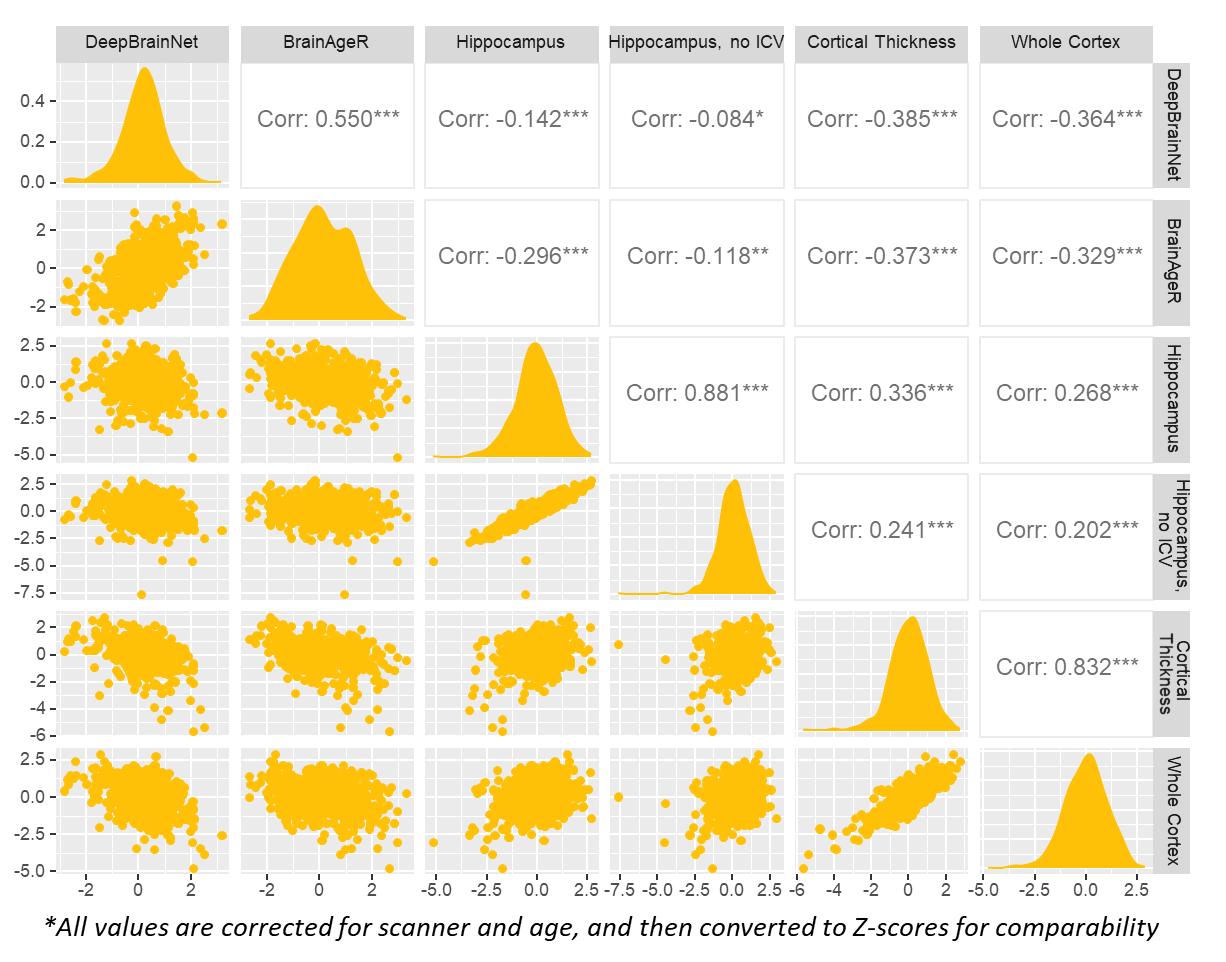


**Supplemental Figure 4**. Comparison of markers of neurodegeneration in Non-Hispanic White participants. Hippocampal volume with and without intracranial volume (ICV) correction have the strongest correlation, followed by AD signature cortical thickness and average whole cortex thickness. The third strongest correlation is between methods of Brain Age Gap (BAG) calculation, followed by Cortical Thickness and BAG_Deep Brain Net_.


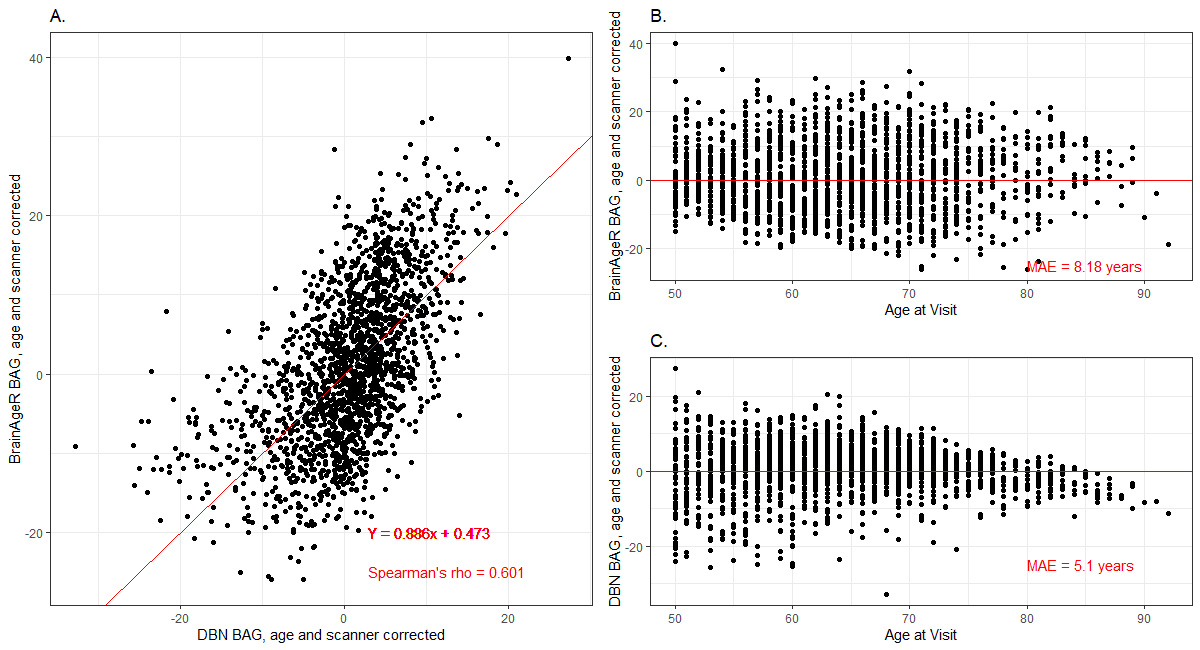


**Supplemental Figure 5.** Validation of Brain Age Gap (BAG) methods. BAG measures calculated via BrainAgeR and Deep Brain Net (DBN) are correlated (A). BrainAgeR has greater variance overall (B) as indicated by both Mean Average Error (MAE) and visual inspection of the plot. DBN is more accurate (C), but demonstrates a systematic underestimation of participant age for participants over the age of approximately 80. This analysis presents all participants, regardless of dementia status.


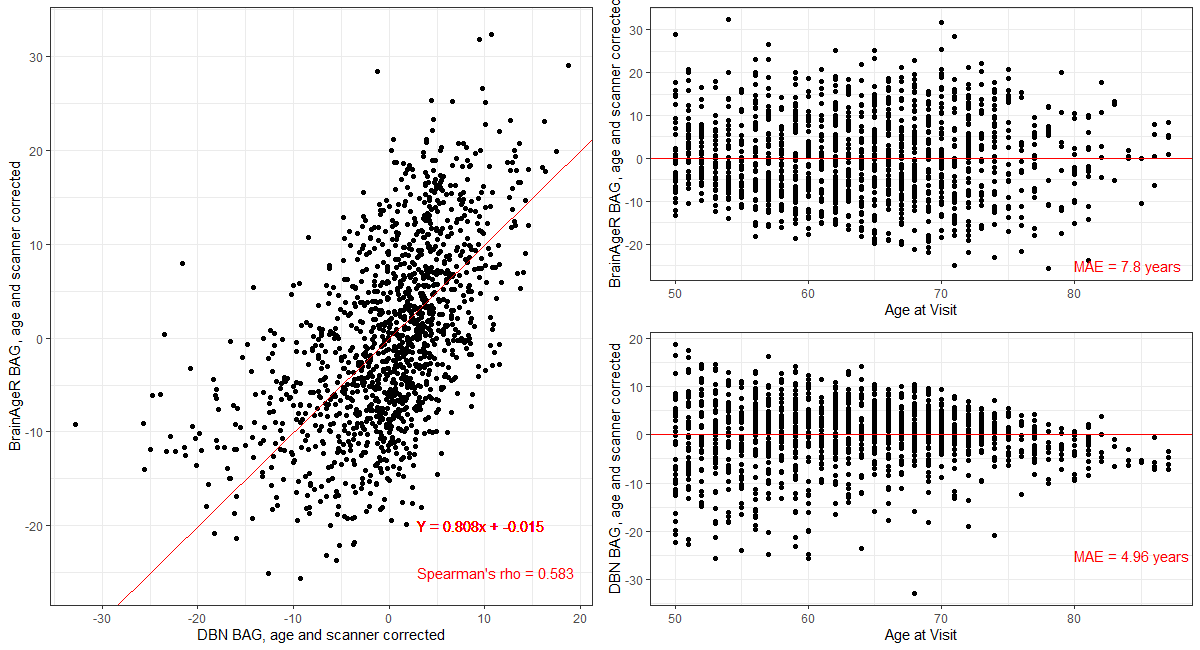


**Supplemental Figure 6.** Validation of Brain Age Gap (BAG) methods in only cognitive normal (CDR = 0) participants. BAG measures calculated via BrainAgeR and Deep Brain Net (DBN) are correlated (A). BrainAgeR has greater variance overall (B) as indicated by both Mean Average Error (MAE) and visual inspection of the plot. DBN is more accurate (C). The accuracy of both models increases slightly with the removal of participants who are cognitively impaired.


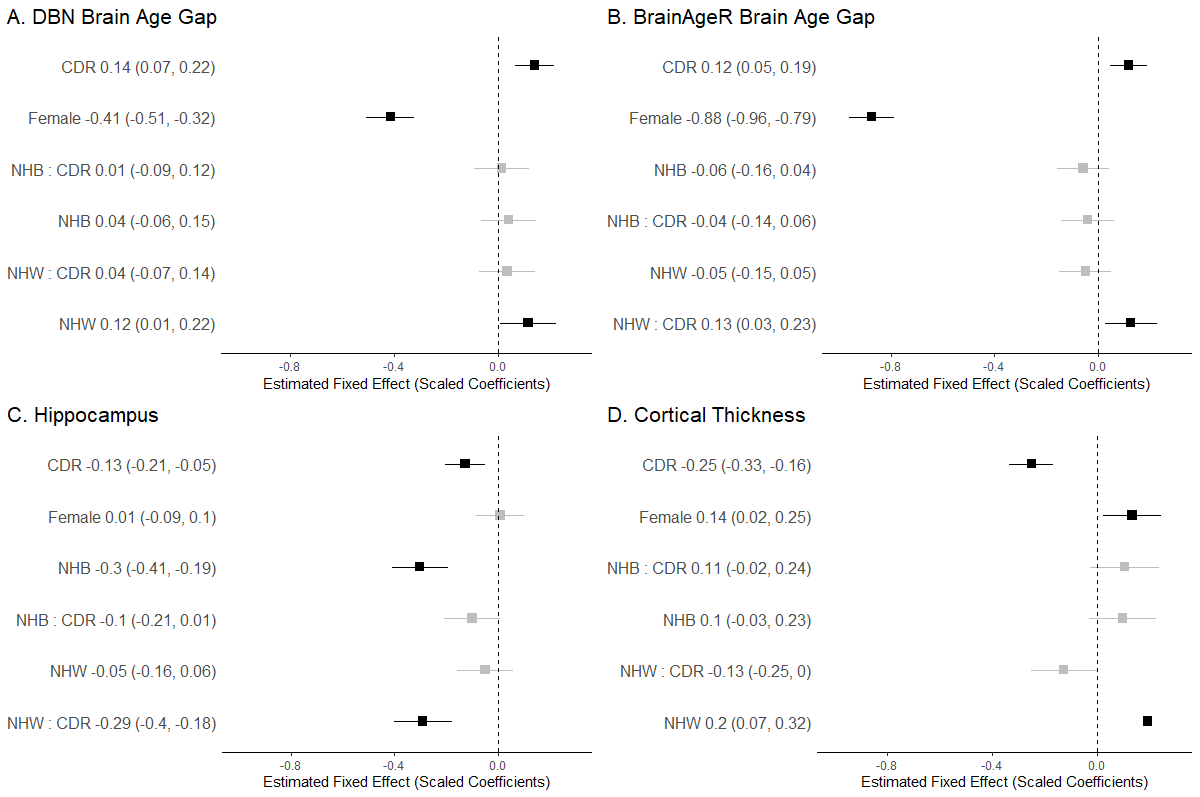


**Supplemental Figure 7. Estimated effects of CDR, racial identity and gender on markers of neurodegeneration.** Brain age gap (BAG) as calculated by DeepBrainNet (DBN) was higher in non-Hispanic White participants (NHW) and lower in females (A). BAG as calculated by BrainAgeR was also lower in females, and there was a significant interaction between NHW and CDR, indicating that BAG increased at a greater rate with increasing dementia severity for NHW than Mexican American (MA) participants (B). Hippocampal volumes were smaller at baseline in non-Hispanic Black (NHB) participants as compared to MA participants, and hippocampal volume declined at a greater rate with increasing dementia severity for NHW participants (C). Cortical thickness was somewhat thicker in both female and NHW participants, although no interactions were present between ethno-racial identity and CDR (D).

**
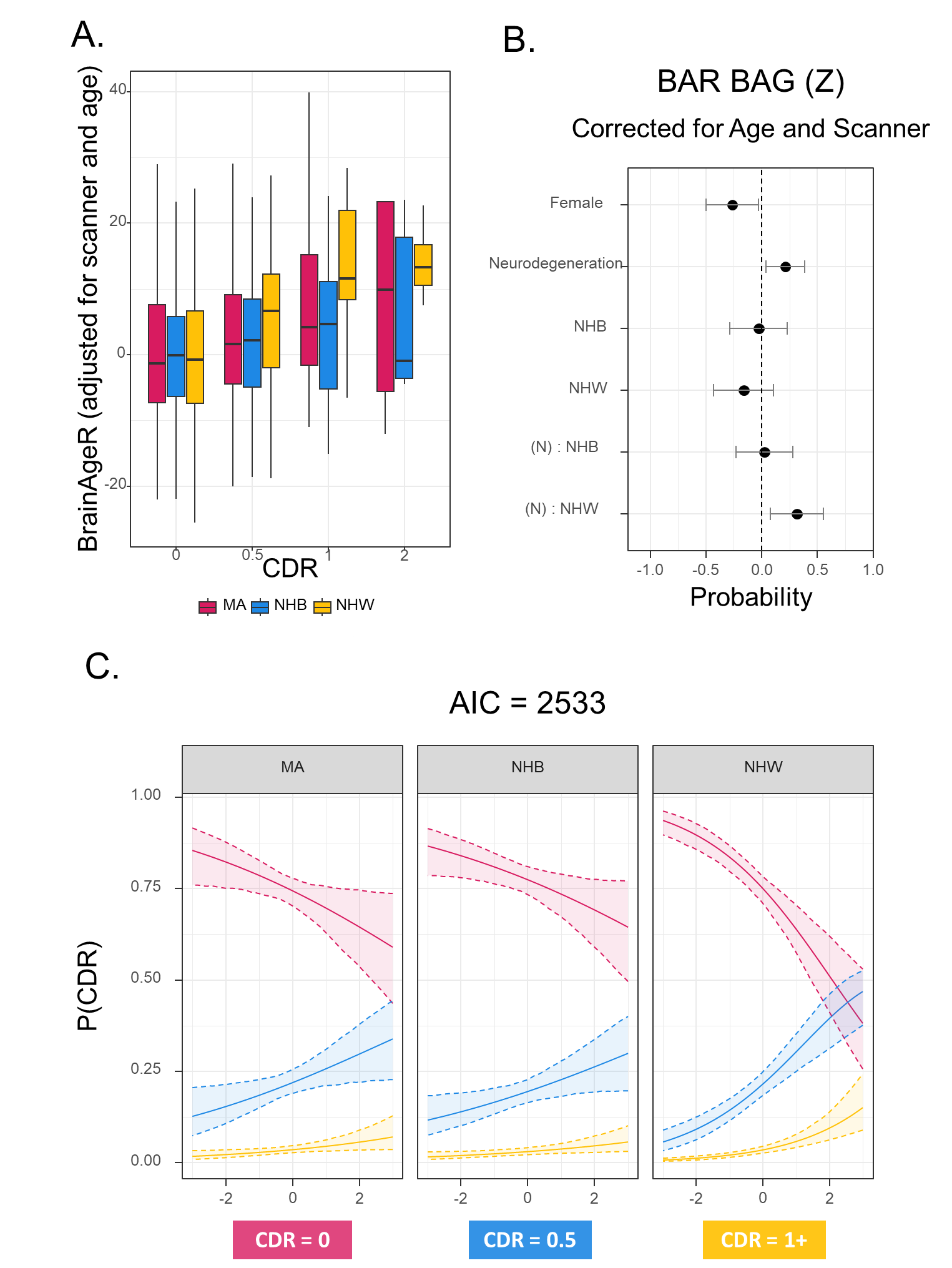
**

**Supplemental Figure 8. Repeated analysis with BrainAgeR as the neuroimaging outcome variable.** (A) We observe the same expected relationship between BAG and CDR as calculated by BrainAgeR as we did by DBN. There is a significant positive relationship between CDR and BAG as calculated by BrainAgeR (*p = 0.00117*). There is a significant CDR by NHW interaction, where BAG increases at a greater rate for NHW than MA with increasing CDR (*p = 0.0136*). Tukey post-hoc test reveals that for MA, MA of CDR ≥ 1 have significantly greater BAG than cognitively normal MA (*Difference = 6.72 years, p_adjusted_ = 0.0296*). NHW of CDR ≥ 1 have significantly greater BAG than NHW of CDR 0.5 (*Difference = 8.4 years, p_adjusted_ = 0.0038*), which have a significantly greater BAG than cognitively normal NHW (*Difference = 4.10 years, p_adjusted_ = 0.0001*). There are no differences by CDR for NHB. (B) In the ordinal regression treating CDR as the outcome variable, we observed a significant main effect of BAG as calculated by BrainAgeR (*p = 0.0160*). There was a significant interaction for NHW with BAG (*p = 0.0232*). (C) Similar to the observed relationship in BAG as calculated with DBN, the probability of being CDR 0 with increasing BAG decreases at a greater rate for NHW than either MA or NHB.


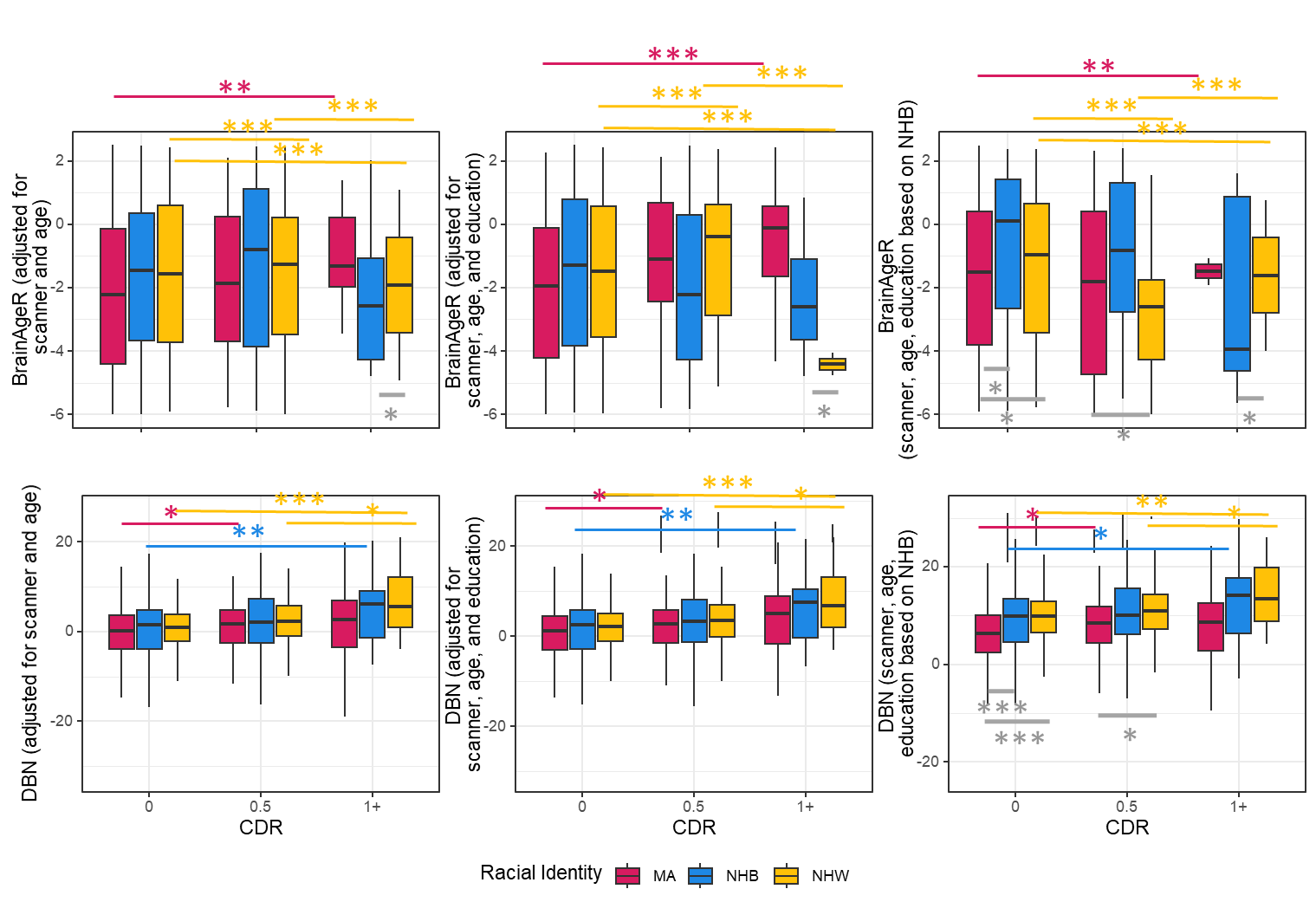


**Supplemental Figure 9. Repeated analysis of Brain Age Gap with considerations for educational attainment.** We re-analyzed the measures of Brain Age Gap (as assessed via either BrainAgeR or DeepBrainNet) and controlled them either for educational attainment across all participants or using two-step correction: we first performed linear regression within the non-Hispanic Black (NHB) cohort only (as the NHB cohort had the most uniform distribution of educational attainment of the three groups), and then we applied the estimated effect of educational attainment to adjust Brain Age Gap for all participants. In both cases, we observed no substantial differences for the relationship between Brain Age Gap and CDR within ethnoracial group. Controlling for educational attainment using the estimated effect of educational attainment in the NHB cohort did result in differences across ethno-racial groups for a given CDR level.


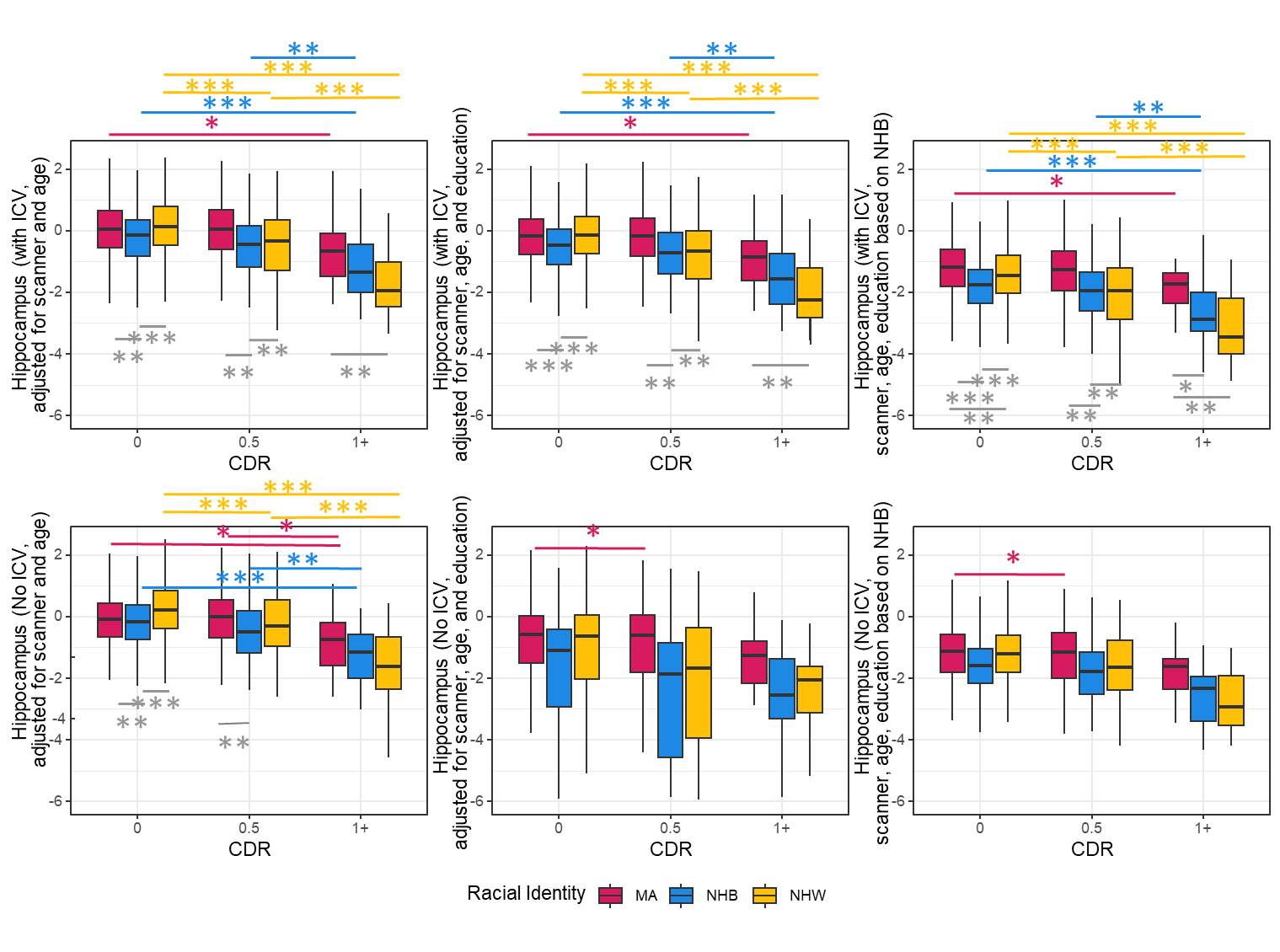


**Supplemental Figure 10. Repeated analysis of Hippocampal Volume with considerations for educational attainment and Intracranial Volume (ICV) correction.** We re-analyzed hippocampal volume and controlled either for educational attainment across all participants or using two-step correction: we first performed linear regression within the non-Hispanic Black (NHB) cohort only (as the NHB cohort had the most uniform distribution of educational attainment of the three groups), and then we applied the estimated effect of educational attainment to adjust hippocampal volume for all participants. Adjustments for educational attainment yielded negligible differences in the relationship between hippocampal volume and CDR. In the original analysis, we performed intracranial volume correction. When we did not perform ICV but left the data uncorrected for educational attainment, we observed results largely similar to the original ICV-corrected results. However, after correcting for educational attainment, we only observed differences within the MA cohort.


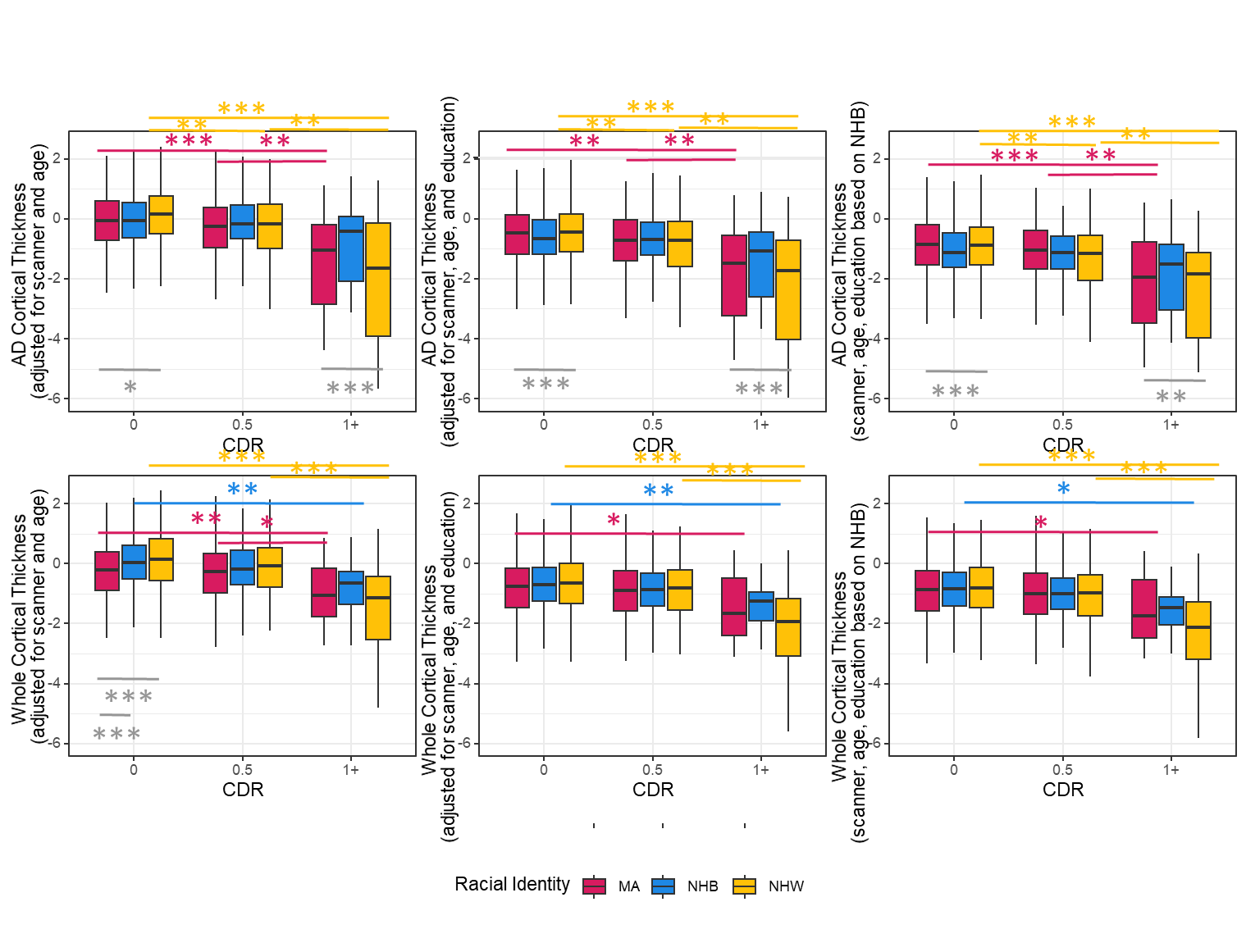


**Supplemental Figure 11. Repeated analysis of Cortical Thickness with considerations for educational attainment and whole cortex thickness.** We re-analyzed cortical thickness and controlled either for educational attainment across all participants or using two-step correction: we first performed linear regression within the non-Hispanic Black (NHB) cohort only (as the NHB cohort had the most uniform distribution of educational attainment of the three groups), and then we applied the estimated effect of educational attainment to adjust cortical thickness for all participants. Adjustments for educational attainment yielded negligible differences in the relationship between cortical thickness and CDR. In the original analysis, we relied on a cortical thickness signature derived to be specific for Alzheimer Disease related changes. We performed a supplemental analysis looking at average thickness across the whole cortex and found a substantially weaker relationship to CDR.


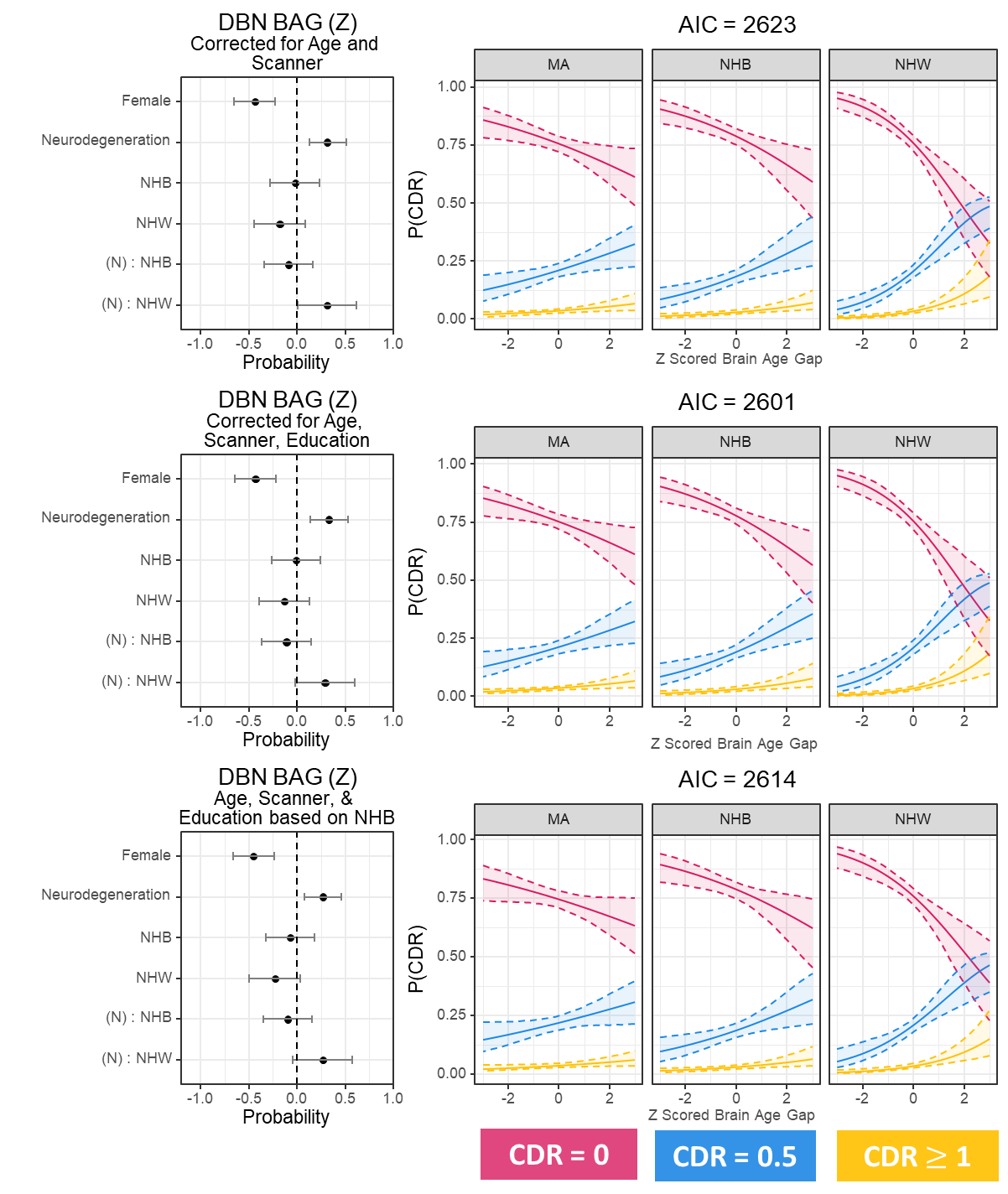


**Supplemental Figure 12. Repeated analysis of Brain Age Gap with considerations for educational attainment.** We re-analyzed Brain Age Gap and controlled it either for educational attainment across all participants or using two-step correction: we first performed linear regression within the non-Hispanic Black (NHB) cohort only (as the NHB cohort had the most uniform distribution of educational attainment of the three groups), and then we applied the estimated effect of educational attainment to adjust Brain Age Gap for all participants. Both forms of educational adjustment yielded minimal differences in probability of having a given CDR assessment.


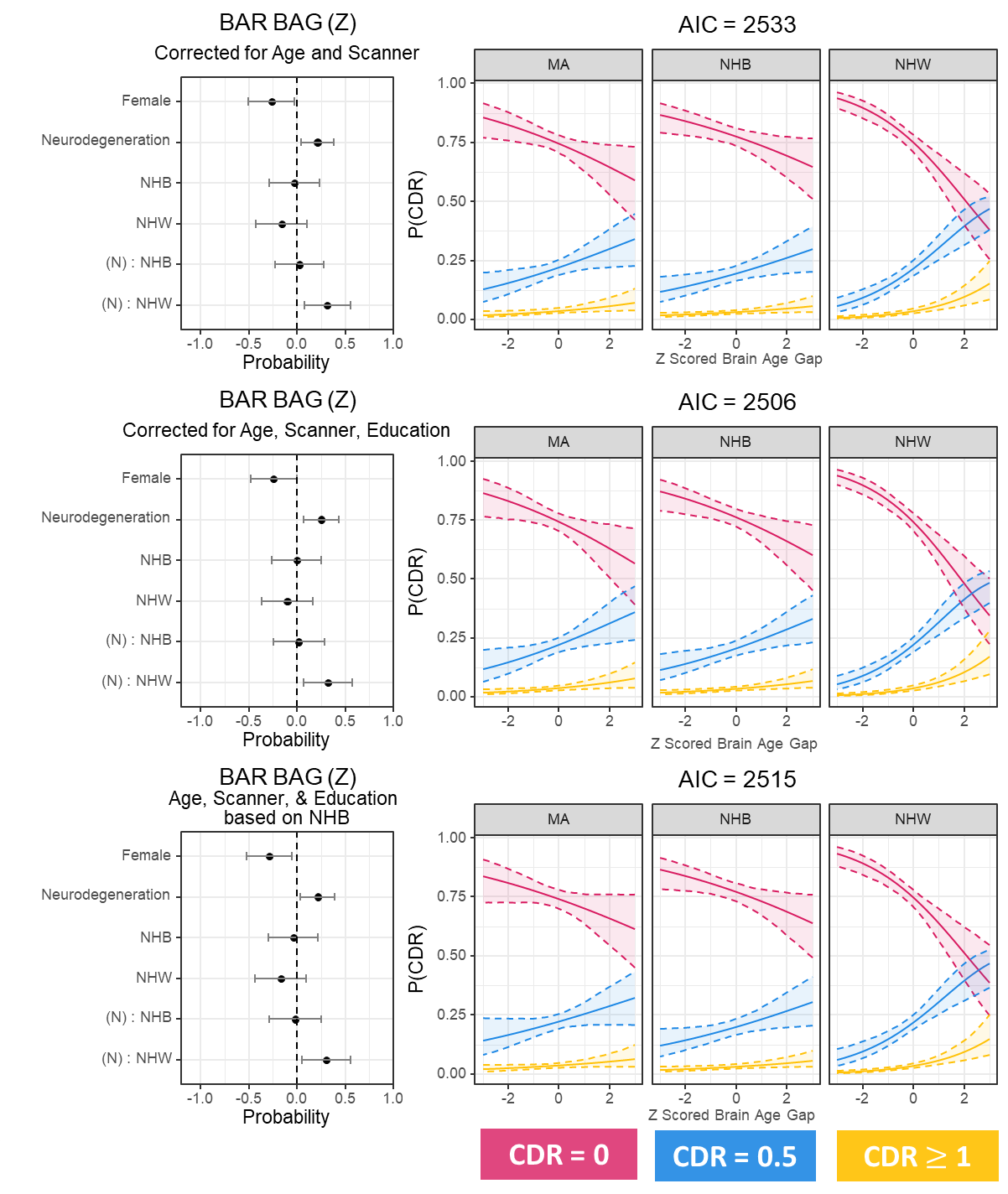


**Supplemental Figure 13. Repeated analysis of Brain Age Gap with considerations for educational attainment.** We re-analyzed Brain Age Gap and controlled it either for educational attainment across all participants or using two-step correction: we first performed linear regression within the non-Hispanic Black (NHB) cohort only (as the NHB cohort had the most uniform distribution of educational attainment of the three groups), and then we applied the estimated effect of educational attainment to adjust Brain Age Gap for all participants. Both forms of educational adjustment yielded minimal differences in probability of having a given CDR assessment.


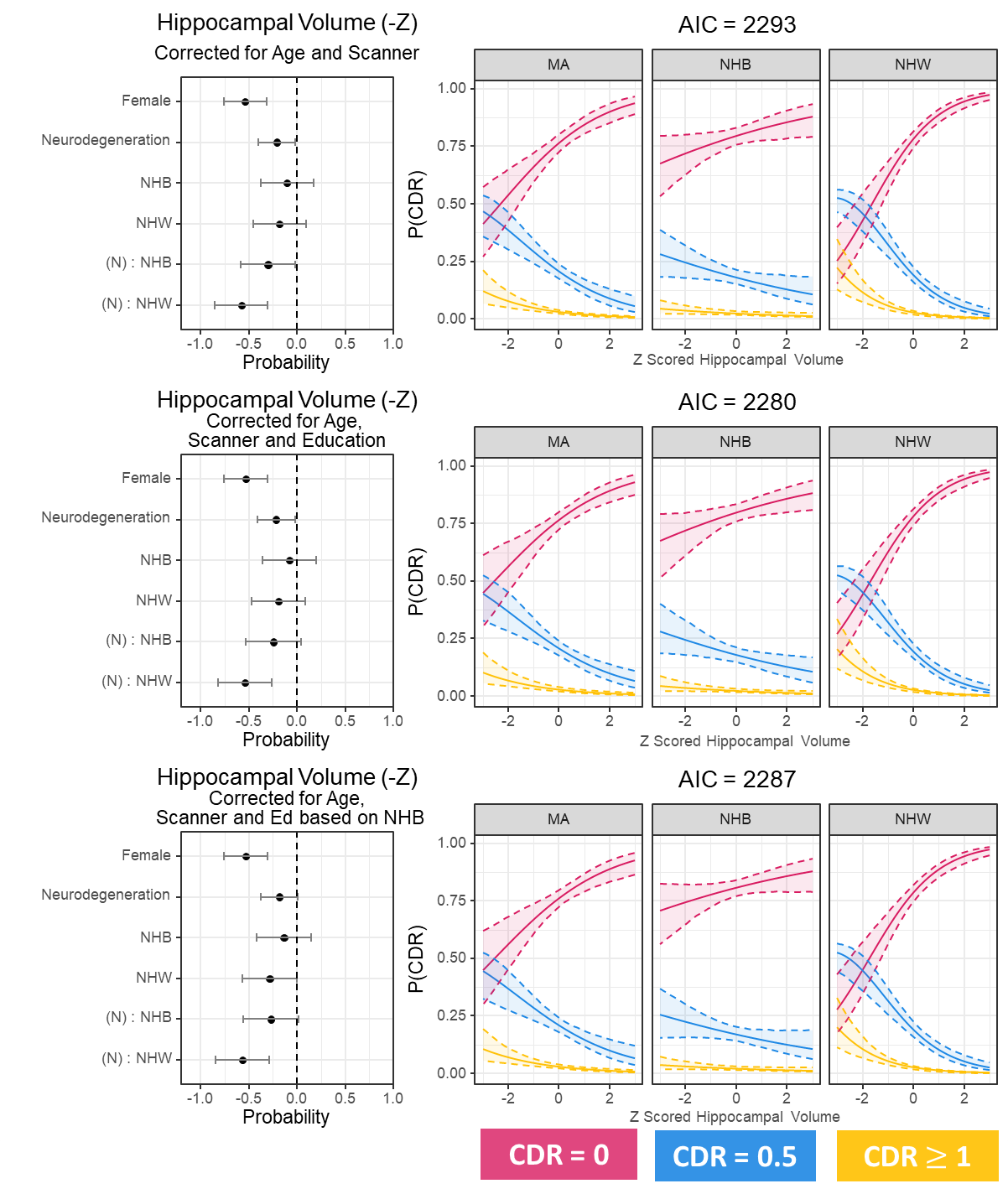


**Supplemental Figure 14. Repeated analysis of Hippocampal Volume with considerations for educational attainment.** We re-analyzed hippocampal volume and controlled it either for educational attainment across all participants or using two-step correction: we first performed linear regression within the non-Hispanic Black (NHB) cohort only (as the NHB cohort had the most uniform distribution of educational attainment of the three groups), and then we applied the estimated effect of educational attainment to adjust hippocampal volujme for all participants. Both forms of educational adjustment yielded minimal differences in probability of having a given CDR assessment.


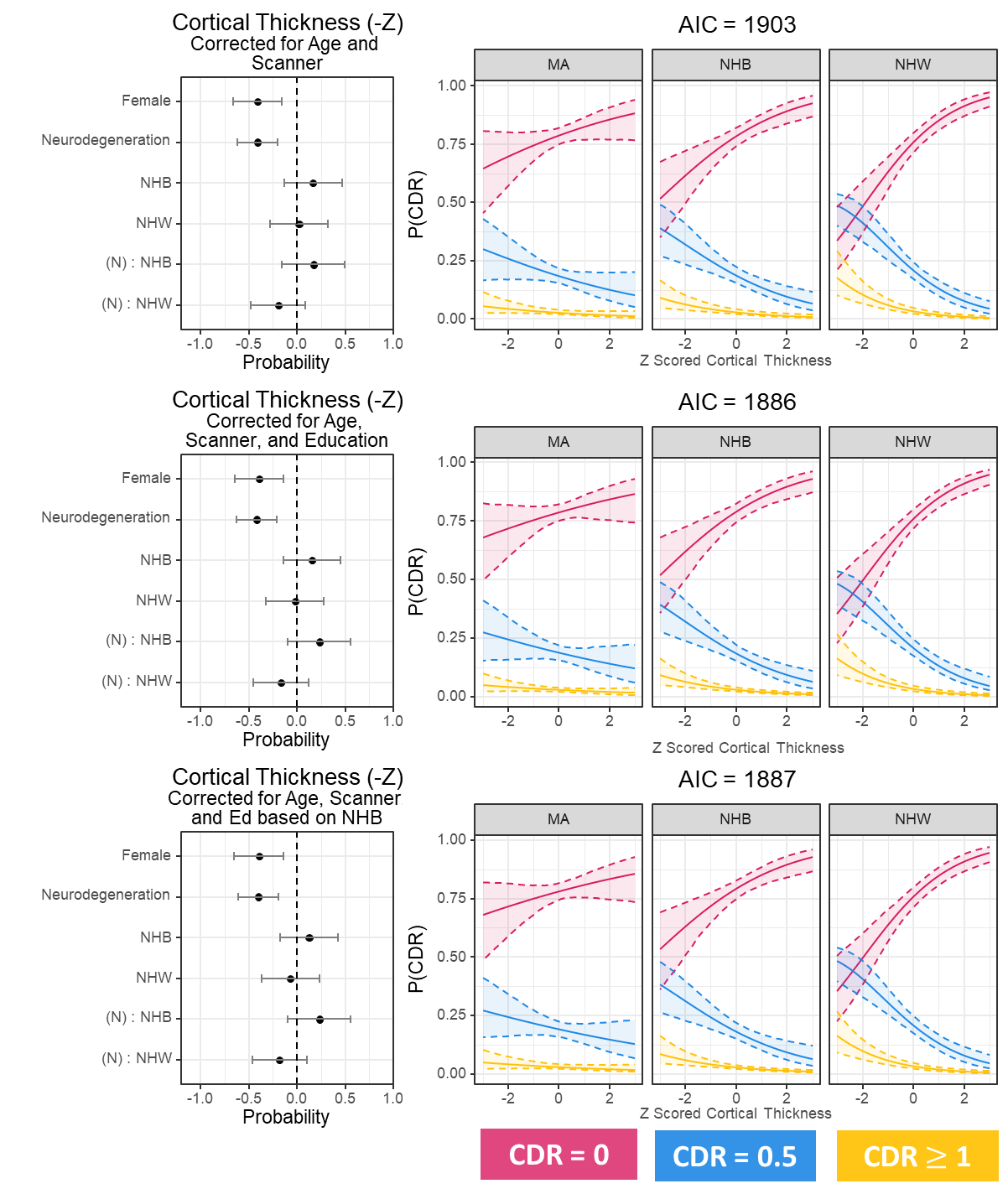


**Supplemental Figure 15. Repeated analysis of Cortical Thickness with considerations for educational attainment.** We re-analyzed the AD signature cortical thickness and controlled it either for educational attainment across all participants or using two-step correction: we first performed linear regression within the non-Hispanic Black (NHB) cohort only (as the NHB cohort had the most uniform distribution of educational attainment of the three groups), and then we applied the estimated effect of educational attainment to adjust hippocampal volujme for all participants. Both forms of educational adjustment yielded minimal differences in probability of having a given CDR assessment.


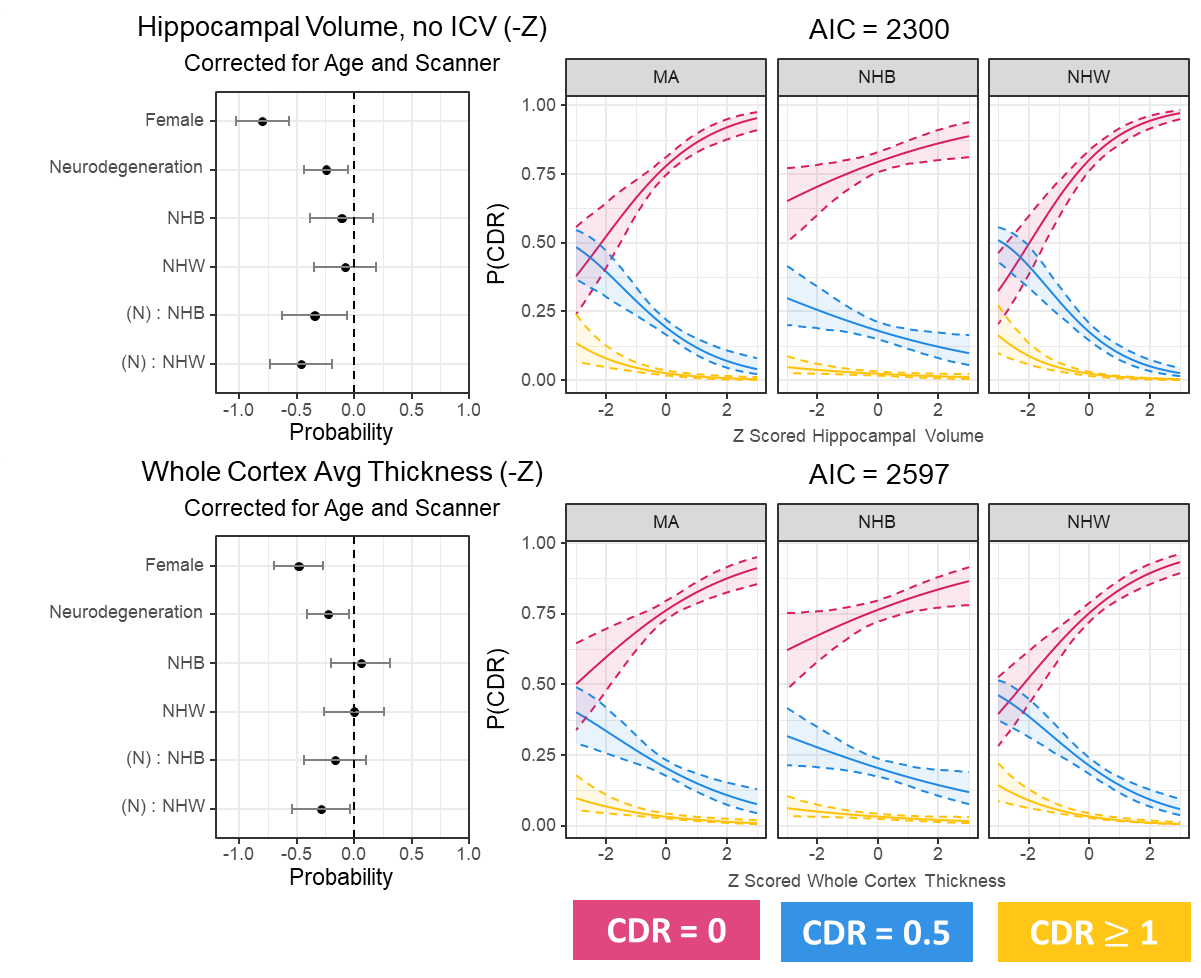


**Supplemental Figure 16. Hippocampal volume absent intracranial volume (ICV) correction and average whole cortex thickness were also considered as possible markers of neurodegeneration that would associate with CDR.** ICV-corrected hippocampal volume and uncorrected hippocampal volume yielded extremely similar results, where there was a significant association between hippocampal volume and CDR, as well as significant ethnoracial group by hippocampal volume interactions. Model fits, as assessed by AIC, were essentially equivalent. We also evaluated the relationship between the whole cortex and CDR in place of the AD signature cortical thickness commonly employed in evaluations of dementia-related clinical changes. The whole cortex was less strongly associated with CDR than the AD signature cortical thickness (as assessed by AIC), and there was a significant interaction between whole cortex thickness and non-Hispanic White (NHW) ethnoracial identity, indicating that, at the group level, whole cortex did not generalize as well as the AD signature cortical thickness.

**HABS-HD Study Team**

HABS-HD MPIs: Sid E O’Bryant, Kristine Yaffe, Arthur Toga, Robert Rissman, & Leigh Johnson; and the HABS-HD Investigators: Meredith Braskie, Kevin King, James R Hall, Melissa Petersen, Raymond Palmer, Robert Barber, Yonggang Shi, Fan Zhang, Rajesh Nandy, Roderick McColl, David Mason, Bradley Christian, Nicole Phillips, Stephanie Large, Joe Lee, Badri Vardarajan, Monica Rivera Mindt, Amrita Cheema, Lisa Barnes, Mark Mapstone, Annie Cohen, Amy Kind, Ozioma Okonkwo, Raul Vintimilla, Zhengyang Zhou, Michael Donohue, Rema Raman, Matthew Borzage, Michelle Mielke, Beau Ances, Ganesh Babulal, Jorge Llibre-Guerra, Carl Hill and Rocky Vig.
